# Oncogenic potential of truncated RXRα during colitis-associated colorectal tumorigenesis by promoting IL-6-STAT3 signaling

**DOI:** 10.1038/s41467-019-09375-8

**Published:** 2019-04-01

**Authors:** Xiaohong Ye, Hua Wu, Luoyan Sheng, Yi-xin Liu, Fang Ye, Mo Wang, Hu Zhou, Ying Su, Xiao-kun Zhang

**Affiliations:** 1 School of Pharmaceutical Sciences, Fujian Provincial Key Laboratory of Innovative Drug Target Research, Xiamen University, 361102 Xiamen, China; 2 Department of Pathology, Soochow University, 215123 Suzhou, China; 3 Sanford Burnham Prebys Medical Discovery Institute, 10901 N. Torrey Pines Road, La Jolla, CA 92037 USA

## Abstract

Retinoid X receptor-alpha (RXRα) is a potent regulator of inflammatory responses; however, its therapeutic potential for inflammatory cancer remains to be explored. We previously discovered that RXRα is abnormally cleaved in tumor cells and tissues, producing a truncated RXRα (tRXRα). Here, we show that transgenic expression of tRXRα in mice accelerates the development of colitis-associated colon cancer (CAC). The tumorigenic effect of tRXRα is primarily dependent on its expression in myeloid cells, which results in interleukin-6 (IL-6) induction and STAT3 activation. Mechanistic studies reveal an extensive interaction between tRXRα and TRAF6 in the cytoplasm of macrophages, leading to TRAF6 ubiquitination and subsequent activation of the NF-κB inflammatory pathway. K-80003, a tRXRα modulator derived from nonsteroidal anti-inflammatory drug (NSAID) sulindac, suppresses the growth of tRXRα-mediated colorectal tumor by inhibiting the NF-κB-IL-6-STAT3 signaling cascade. These results provide new insight into tRXRα action and identify a promising tRXRα ligand for treating CAC.

## Abstract (web summary)

The truncated form of retinoid X receptor-alpha is found in cancer. Here, the authors show that this mutant receptor promotes colorectal tumorigenesis by activation of NF-κB in myeloid cells, leading to subsequent IL-6-induced STAT3 activation in intestinal epithelial cells, which can be suppressed by ligand K-80003.

## Introduction

Retinoid X receptor α (RXRα), a master member of the nuclear receptor superfamily, regulates a broad spectrum of cellular processes under physiological and pathophysiological conditions^1–3^. Targeted disruption of RXRα gene leads to preneoplastic lesions in prostate^4^, alopecia, epidermal interfollicular hyperplasia, keratinocyte hyperproliferation, and aberrant terminal differentiation in the skin^5^ and the development of cervical malignant lesions^6^. Altered expression and modification of RXRα is also implicated in the development of a number of malignancies^1,3,7–9^. RXRα binding to promyelocytic leukemia (PML)/RARα is absolutely required for the development of acute PML in transgenic mice, revealing its oncogenic potential when acting inappropriately^10,11^. Interestingly, RXRα is proteolytically cleaved in cancer cells^12–26^, implying that aberration in RXRα signaling by limited proteolysis plays a role in cancer development. Consistent with its role in cancer development, RXRα is one of the most important targets for the development of pharmacologic intervention and therapeutic applications^1,7,27–30^. Notably, Targretin® was approved for treating human cutaneous T cell lymphoma^27^. However, the therapeutic potential of RXRα modulators remains to be explored, which requires our further understanding of its role in tumorigenesis.

Chronic inflammation is an important contributor to increased risk of cancer^31,32^. RXRα and ligands are potent regulators of inflammatory responses. RXRα is highly expressed in all inflammatory cell types^33^. Analysis of macrophage-specific RXRα knockout mice revealed a critical role of RXRα in regulating macrophage functions and inflammatory responses, including the upregulation of chemokine expression and reduction of antiviral responses in myeloid cells^33–35^. RXRα compounds also regulate various inflammatory pathways in different cell types^36–40^. Interestingly, certain anti-inflammatory agents, such as docosahexaenoic acid^41^, R-etodolac^42^, and sulindac^25^, serve as RXRα ligands, further supporting the role of RXRα in regulating inflammatory responses. However, the underlying mechanisms by which RXRα and ligands act, especially whether and how they mediate and modulate the causal link between inflammation and cancer remain obscure.

RXRα, like other nuclear receptors, consists of three distinct domains: an N-terminal A/B region, a DNA-binding domain (DBD), and a C-terminal ligand-binding domain (LBD)^1,2,8^. The presence of well-conserved DBD in RXRα and other nuclear receptors led to the discovery that members of the nuclear receptor superfamily serve as ligand-dependent nuclear transcription factors^2^. Subsequent studies, however, have revealed that RXRα and other nuclear receptors could also act independently of their DNA binding and transcription function^9,43^. Orphan nuclear receptor Nur77 translocates from the nucleus to the cytoplasm where it acts at mitochondria to promote apoptosis^44,45^ and mitophagy^46^, whereas steroid hormone receptors interact with the p85α subunit of phosphoinositide 3-kinase (PI3K) in the cytoplasm to modulate the PI3K survival pathway^47,48^. RXRα is predominantly nuclear but can migrate to the cytoplasm in response to inflammation^23,25,49,50^. We previously reported that RXRα is proteolytically cleaved in cancer cells, resulting in production of a truncated RXRα (tRXRα) that lacks a portion of its N-terminal A/B domain^25^. Unlike full-length RXRα, tRXRα is predominantly cytoplasmic in response to inflammatory cytokine tumor necrosis factor-α (TNFα), interacting with the p85α to activate the PI3K/AKT pathway^25^. Thus tRXRα plays a critical role in mediating the survival effect of inflammatory signaling through its non-transcriptional action. The role of tRXRα was further illustrated by our finding that tRXRα activity was inhibited by sulindac, a nonsteroidal anti-inflammatory drug (NSAID)^51^, and analogs^21,25,52^.

Colorectal cancer is closely associated with chronic inflammation^53,54^, and regular use of NSAIDs lowers the mortality from colorectal cancer^55^. RXRα downregulation^38^ or malfunction of RXRα due to phosphorylation^56^ is associated with the development of colorectal cancer. To establish the tumorigenic effect of tRXRα in the development of inflammation-associated cancer and the therapeutic potential of targeting tRXRα-mediated inflammatory signaling pathways, we have generated tRXRα transgenic mice. Here we report our characterization of the tRXRα transgenic mice with respect to its tumorigenic effects in the development of colitis-associated colon cancer (CAC), the underlying molecular mechanism, and the therapeutic significance.

## Results

### Tumorigenic effect of tRXRα in mouse colon cancer model

**Fig. 1:**
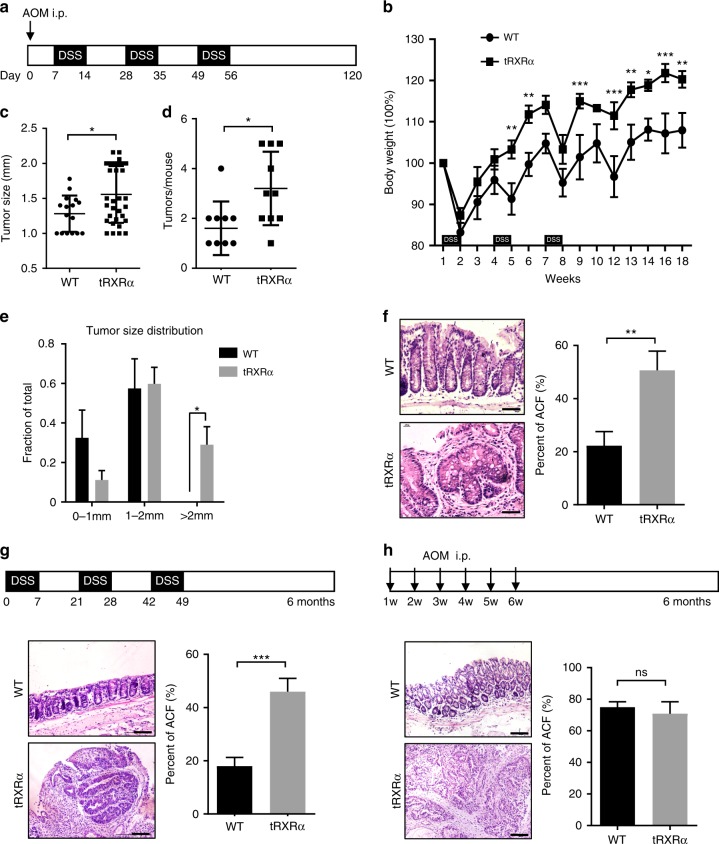
Tumorigenic effect of truncated retinoid X receptor α (tRXRα) in colitis-associated colon cancer mouse model. **a** Schematic representation of the azoxymethane (AOM)/dextran sodium sulfate (DSS) procedure. i.p. intraperitoneal. **b** Body weight curve of control wild-type (WT) and *Tg-tRXRα* mice during the course of AOM/DSS colitis. Data are mean ± SEM (standard error of the mean, *n* = 7), two-way analysis of variance (ANOVA). **c** Average tumor size. Each dot represents a tumor. Data are mean ± SD (standard deviation, *n* = 10), *t* test. **d** Average tumor number. Each dot represents the tumor number of one individual mouse. Data are mean ± SD (*n* = 10), *t* test. **e** Tumor size distribution. Data are mean ± SEM (*n* = 10), two-way ANOVA. **f** Colon tumor sections from mice treated with AOM/DSS at 120 days were microscopically analyzed and examined for microscopic aberrant crypt foci (ACF) after hematoxylin and eosin (H&E). Scale bars, 50 μm. Data are mean ± SEM (*n* = 10/8), *t* test. **g** Role of tRXRα in promoting DSS-induced colorectal carcinogenesis. Schematic representation of the 2.5% DSS procedure (top panel). Colon sections from mice treated with DSS for 6 months were microscopically analyzed and examined for microscopic ACF after H&E. Scale bars, 100 μm. Data are mean ± SEM (*n* = 10), *t* test. **h** Role of tRXRα in promoting AOM-induced colorectal carcinogenesis. Schematic representation of the AOM (10 mg/kg) procedure (top panel). Colon sections from mice treated with AOM for 6 months were microscopically analyzed and examined for microscopic ACF after H&E. Scale bars, 100 μm. Data are mean ± SEM (*n* = 6); ns not significant. *P < 0.05, **P < 0.01, ***P < 0.001

To test the tumorigenic effect of tRXRα in the development of inflammation-associated cancer, we generated a *loxP-tRXRα* (*tRXRα*^*flox*^) transgenic mouse line that contained loxP-flanked SV40 poly A sequences between CAG promoter and tRXRα cDNA sequences (Supplementary Fig. 1a). The loxP-tRXRα mice were crossed with the CMV-Cre mice expressing Cre recombinase under the control of cytomegalovirus (CMV) promoter to produce transgenic tRXRα mice (Tg-tRXRα) (Supplementary Fig. 1b) that express various levels of tRXRα in all the tissues examined (Supplementary Fig. 1c). *Tg-tRXRα* mice, when compared to control wild-type mice, showed certain aberrant crypts in the colon of *Tg-tRXRα* mice (21-month old; Supplementary Fig. 1d). To address the inflammatory effect of tRXRα, *Tg-tRXRα* and control *tRXRα*^*flox*^ mice were subjected to a single injection with the colonotropic mutagen azoxymethane (AOM) followed by three cycles of treatment with the luminal toxin dextran sodium sulfate (DSS) (Fig. 1a), a standard CAC mouse model^57^. We found that *Tg-tRXRα* mice lost less body weight than control mice upon AOM/DSS treatment (Fig. 1b), suggesting that tRXRα expression is involved in the proliferation and survival of colonic epithelial cells. When tumor load was analyzed, *Tg-tRXRα* mice showed increased tumor size (1.556 ± 0.07171 mm, *N* = 32) as compared to control mice (1.281 ± 0.06477 mm, *N* = 16) (Fig. 1c). The number of macroscopic tumors in *Tg-tRXRα* mice (3.2 ± 0.4667 tumors per mouse, *N* = 10) was also increased by two-fold when compared to control animals (1.6 ± 0.3399 tumors per mouse, *N* = 10) (Fig. 1d). As a result, *Tg-tRXRα* mice exhibited a high frequency of larger tumors than control mice (Fig. 1e). Histological analyses confirmed more aberrant crypt foci and hyper-plastic crypts in *Tg-tRXRα* mice (Fig. 1f). These data suggested that tRXRα may play a role in promoting the development of CAC.

To further characterize the role of tRXRα, *Tg-tRXRα* mice were treated with either DSS or AOM. When treated with three cycles of DSS, we found that 2 of the 10 *Tg-tRXRα* mice developed at least 1 tumor, while none of 12 control mice had tumor under the same treatment (Fig. 1g; Table 1). Histological examination confirmed the presence of adenomas in the colon of *Tg-tRXRα* mice (Fig. 1g). AOM is a potent carcinogen that causes a high incidence of colon cancer in rodents^57,58^. Although none of the 6 control mice showed tumor 6 months after repetitive intraperitoneal treatment of AOM, *Tg-tRXRα* mice were much more sensitive to AOM treatment with 2 of the 6 *Tg-tRXRα* mice developing adenoma tumors (Fig. 1h; Table 1), as revealed by histological examination (Fig. 1h), suggesting that tRXRα is involved in modulating the initial response to AOM. Taken together, these data demonstrate that tRXRα acts at different levels to modulate the development of colorectal tumor.

**Table 1:** Transgenic expression of tRXRα promotes colorectal carcinogenesis

Model	DSS alone				AOM alone		
Group of mice	WT	tRXRα			WT	tRXRα	
Number of mice	12	8	1	1	6	4	2
Tumors of mouse	0	0	2	1	0	0	1
Mice with tumours	0		20%		0	33.4%	
*P* value	0.05211				0.0607		

*AOM* azoxymethane, *DSS* dextran sodium sulfate, *tRXRα* trunacted retinoid X receptor α, *WT* wild type

### Activation of STAT3 in colorectal tumors from *Tg-tRXRα* mice

**Fig. 2:**
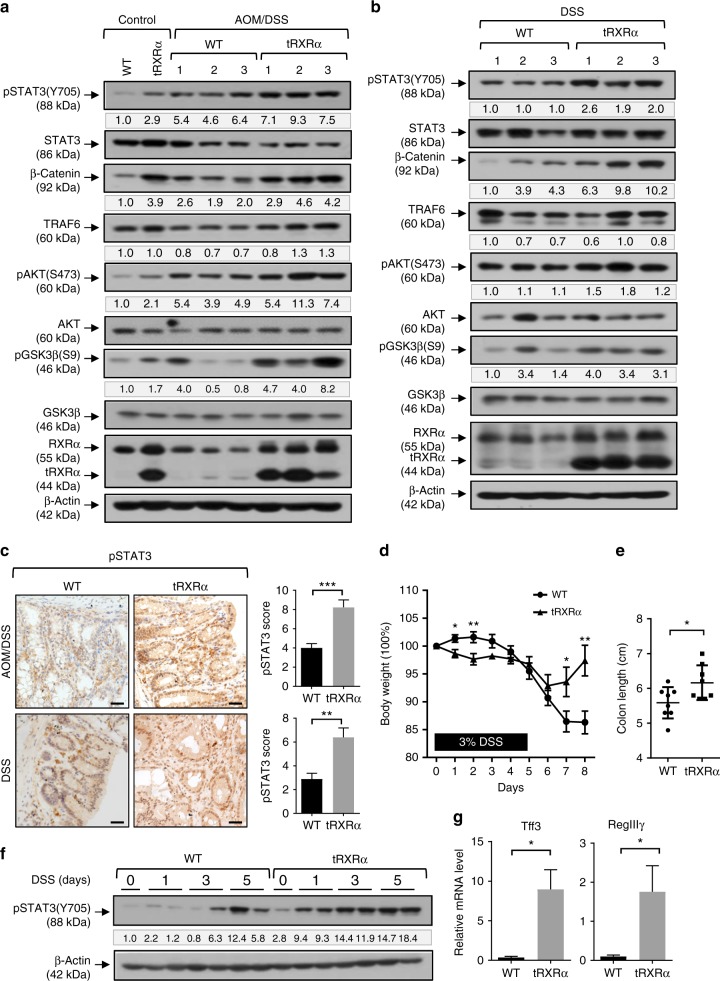
Activation of signal transducer and activator of transcription factor 3 in colorectal tumors from Tg-tRXRα mice. **a**, **b** Colon lysates from mice treated with azoxymethane (AOM)/dextran sodium sulfate (DSS) for 120 days (**a**) or DSS for 6 months (**b**) were analyzed by immunoblotting. The bands are quantified with Image J and normalized to β**-**actin. **c** Colon sections from mice treated with AOM/DSS for 120 days or DSS for 6 months were stained with pSTAT3 antibody and analyzed the staining score. Scale bars, 25 μm. Data are mean ± SEM (*n* = 9), *t* test. **d** Body weight curve of control and *Tg-tRXRα* mice during the course of acute DSS-induced colitis. Data are mean ± SEM (*n* = 8), two-way analysis of variance. **e** Colon length from mice after the course of acute DSS-induced colitis. Data are mean ± SD (*n* = 8), *t* test. **f** Colonic lysates prepared from mice treated with DSS for the indicated time were analyzed by immunoblotting. **g** RegIIIγ and Tff3 mRNA expression in mice treated with DSS for two days was analyzed by quantitative reverse transcriptase–PCR. Data are mean ± SEM (*n* = 3), *t* test. *P < 0.05, **P < 0.01, ***P < 0.001. For immunoblotting, one of three or four similar experiments is shown

To explore the mechanism by which tRXRα impacts tumorigenesis, we determined the activation of signaling pathways critical for colorectal carcinogenesis in tumor tissues from *Tg-tRXRα* mice. In agreement with our previous finding that tRXRα could activate the PI3K/AKT pathway^25^, the level of pAKT was elevated in tumor tissues from *Tg-tRXRα* mice treated with either AOM/DSS (Fig. 2a) or DSS alone (Fig. 2b). The A/B domain of RXRα was shown to interact with β-catenin to trigger its degradation^59^. Transgenic expression of tRXRα that lacks a large portion of the A/B domain resulted in elevated level of β-catenin independently of the treatment with AOM/DSS (Fig. 2a) or DSS (Fig. 2b), likely due to the dominant-negative effect of tRXRα. Interestingly, glycogen synthase kinase (GSK)-3β phosphorylation at Serine 9, which is a substrate of AKT^60^, was also elevated in *Tg-tRXRα* mice. As phosphorylation of GSK-3β could inhibit its activity known to induce β-catenin degradation^60^, enhanced GSK-3β phosphorylation may also play a role in the accumulation of β-catenin in *Tg-tRXRα* mice. Emerging evidence has now revealed a critical role of signal transducer and activator of transcription factor 3 (STAT3) activation in CAC tumorigenesis^61,62^. We found that the level of phosphorylated STAT3 was consistently elevated in *Tg-tRXRα* mice even in the absence of treatment (Fig. 2a). In agreement with previous reports^62^, AOM/DSS treatment resulted in STAT3 activation in control mice. However, the activation was significantly enhanced in *Tg-tRXRα* mice (Fig. 2a). Tg-tRXRα mice also exhibited higher STAT3 activation upon DSS treatment compared to control mice (Fig. 2b). Enhanced STAT3 activation by tRXRα was confirmed by immunohistochemical staining in tumor tissues from *Tg-tRXRα* and control mice treated with AOM/DSS or DSS (Fig. 2c). These data revealed for the first time an important role of tRXRα in the activation of STAT3 during colorectal tumorigenesis.

STAT3 could be activated in acute DSS-induced colitis, protecting DSS-induced colonic epithelium injury^61,62^. We next determined whether tRXRα expression impacted the activation of STAT3 in the acute DSS-induced colitis model that uses a single 5-day course of DSS. *Tg-tRXRα* and control mice were treated with 3% DSS in drinking water for 5 days followed by 4 days of recovery in normal drinking water. Starting from day 1 of DSS treatment, *Tg-tRXRα* mice lost significant more weight than control animals but rapidly regained body weight after removal of DSS from the drinking water (Fig. 2d). In contrast, control mice continued to lose weight when DSS was removed from the drinking water for 2 more days. As a result, colon length of *Tg-tRXRα* mice was significantly longer compared to control mice (Fig. 2e). Consistent with the initial rapid body weight loss, *Tg-tRXRα* mice showed more epithelial injury and crypt incomplete 2 days after DSS treatment (Supplementary Fig. 2a). When STAT3 activation was assessed, we found that DSS activation of STAT3 occurred much earlier in *Tg-tRXRα* mice than in control mice. Upregulation of STAT3 phosphorylation was seen 3 days after DSS treatment in control mice. However, enhanced STAT3 activation was clearly seen 1 day after DSS treatment in *Tg-tRXRα* mice and continued to increase after 3- or 5-day treatment (Fig. 2f). The expression of the tissue-protective factors TFF3 and Reglllγ, the downstream targets of STAT3, which are known to mediate the protective effect of STAT3 on intestinal injury during colitis^62^, was also significantly elevated in *Tg-tRXRα* mice (Fig. 2g). Body weight recovery from DSS treatment is a result of hyperproliferation of epithelial cells caused by STAT3 activation^61^. In line with their rapid recovery from body weight loss, *Tg-tRXRα* mice exhibited less severe epithelial cell damage, loss of crypts, and ulceration compared with control mice after the acute DSS-induced colitis protocol (Supplementary Fig. 2b). Immunohistochemical staining revealed enhanced Ki-67 and proliferating cell nuclear antigen (PCNA) staining in the crypts of *Tg-tRXRα* mice (Supplementary Fig. 2c, d). These results suggested that STAT3 activation might play a role in tRXRα-induced protection of intestinal cells from DSS-induced injury and promotion of intestinal epithelial cell survival and proliferation.

### Increased infiltration of inflammatory cells in tRXRα tumor

**Fig. 3:**
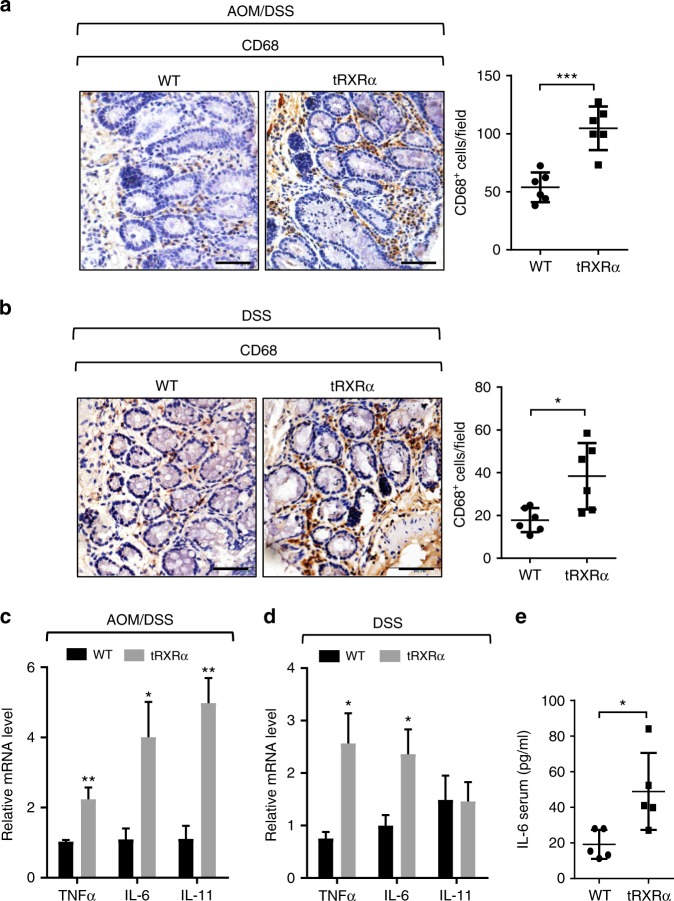
Increased infiltration of inflammatory cells and cytokine production in *Tg-tRXRα* tumor. **a**, **b** Colonic sections from mice treated with azoxymethane (AOM)/dextran sodium sulfate (DSS) (**a**) or DSS (**b**) were stained with CD68 antibody to detect macrophages and analyzed for the number of CD68-positive cells per field (scale bars, 50 μm). Each dot represents a mouse, 10 fields were counted per mouse. Data are mean ± SD (*n* = 6), *t* test. **c**, **d** Relative mRNA expression levels of the indicated genes in colon of mice treated with AOM/DSS (**c**) or DSS (**d**) were determined by quantitative reverse transcriptase–PCR. Data are mean ± SEM (*n* = 4), *t* test. **e** Serum levels of interleukin-6 from mice treated with 3% DSS for 2 days were measured by enzyme-linked immunosorbent assay. Each dot represents a mouse. Data are mean ± SD (*n* = 5), *t* test. *P < 0.05, **P < 0.01, ***P < 0.001

Our finding that STAT3 was strongly activated in *Tg-tRXRα* tumors prompted us to study the mechanism by which tRXRα promotes STAT3 activation. Cytokines especially interleukin (IL)-6 and IL-11 through their respective receptors can phosphorylate and activate STAT3^61,62^. Our immunohistochemical study revealed much more pronounced staining of the macrophage marker CD68 in lamina propria of tumor tissue from *Tg-tRXRα* mice than that from control mice treated with AOM/DSS (Fig. 3a), indicating an increased infiltration of inflammatory cells in *Tg-tRXRα* mice. In tumor developed from *Tg-tRXRα* mice subjected to DSS treatment solely, a significant number of infiltrated inflammatory cells was also detected (Fig. 3b). When the expression levels of cytokines were examined in mice treated with AOM/DSS, we found increased mRNA expression levels of IL-6, IL-11, and TNFα in tumor tissues from *Tg-tRXRα* mice (Fig. 3c). DSS-treated *tRXRα* mice also showed a noticeable increase in mRNA expression of IL-6 and TNFα but not of IL-11 (Fig. 3d). In contrast, *Tg-tRXRα* mice treated with AOM did not show any apparent effect on macrophage infiltration (Supplementary Fig. 3a) and the expression of IL-6, IL-11, and TNFα, although cyclin D2 expression was increased (Supplementary Fig. 3b). IL-6 produced by lamina propria myeloid cells can protect intestinal epithelial cells from apoptosis through activation of STAT3^62^. We found that *Tg-tRXRα* mice produced high level of serum IL-6 2 days after DSS treatment (Fig. 3e), in agreement with their rapid recovery from body weight loss induced by DSS treatment (Fig. 2d) and enhanced STAT3 activation (Fig. 2f). Together, these results revealed an important role of tRXRα in modulating inflammatory microenvironment and cytokine production, which likely accounts for its activation of STAT3 during colitis.

### Myeloid tRXRα expression is necessary for STAT3 activation

**Fig. 4:**
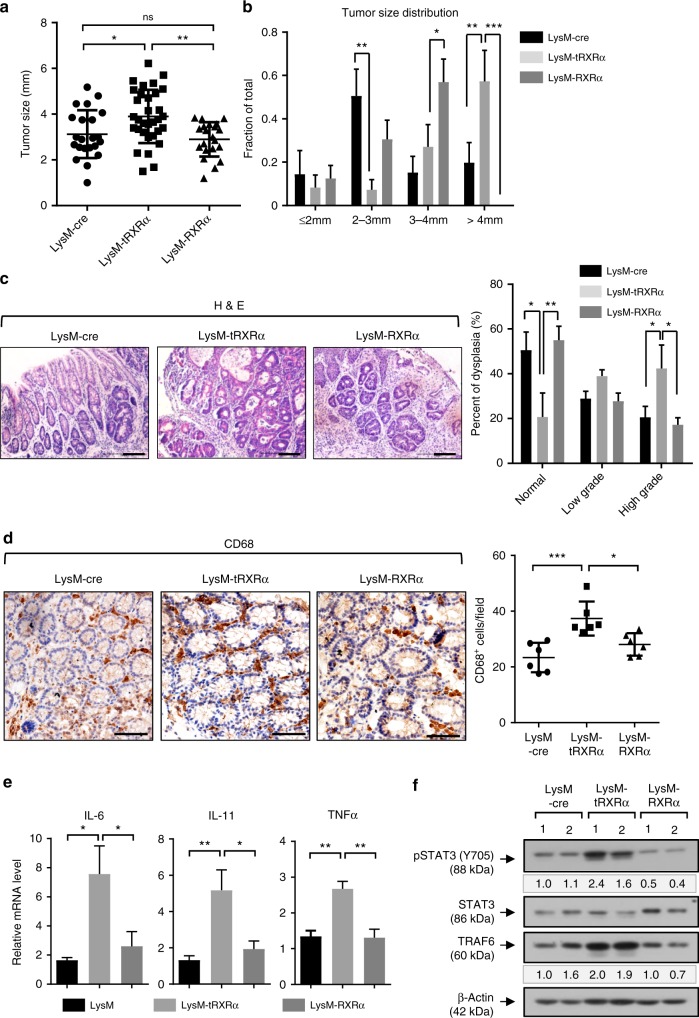
Myeloid-specific truncated retinoid X receptor α expression is necessary for signal transducer and activator of transcription factor 3 activation and colorectal carcinogenesis. **a** Tumor size from mice treated with azoxymethane (AOM)/dextran sodium sulfate (DSS) for 4 months. Each dot represents a tumor. Data are mean ± SD (*n* = 6), one-way analysis of variance (ANOVA). **b** Tumor size distribution. Data are mean ± SEM (*n* = 6), two-way ANOVA. **c** Colon tumor sections from mice treated with AOM/DSS for 4 months were stained with hematoxylin and eosin, Scale bars, 100 μm, and classified into normal, low-grade, and high-grade lesions based on histological analysis (*n* = 6). Data are mean ± SEM (*n* = 6), two-way ANOVA. **d** Representative CD68 immunostaining image of colonic sections from mice treated with AOM/DSS (scale bars, 50 μm) and the score of the number of CD68-positve cells per field. Each dot represents a mouse, and 10 fields were counted per mouse. Data are mean ± SD (*n* = 6), one-way ANOVA. **e** Relative mRNA expression levels of the indicated genes in colon of mice treated with AOM/DSS were determined by quantitative reverse transcriptase–PCR. Data are mean ± SEM (*n* ≥ 4). one-way ANOVA. **f** Colon lysates from mice treated with AOM/DSS were analyzed by immunoblotting. The bands are quantified with Image J and normalized to β**-**actin. One of three similar experiments is shown. *P < 0.05, **P < 0.01, ***P < 0.001

Our observation that *Tg-tRXRα* mice could promote macrophage infiltration and cytokine production prompted us to determine the effect of tRXRα expression in myeloid cells. Thus we crossed *tRXRα*^*flox*^ mice with *LysM-Cre* mice that express Cre in myeloid cells^63^. For comparison, *RXRα*^*flox*^ mice were also crossed with *LysM-Cre* mice. These mice were initially analyzed for tumorigenesis using the AOM/DSS protocol. When *LysM-tRXRα* mice were analyzed, we found that the average tumor size was much larger in this group of mice when compared to *LysM-cre* mice (Fig. 4a). Overexpression of RXRα in myeloid cells, however, did not apparently affect tumor load as *LysM-RXRα* mice showed similar tumor size and multiplicity with *LysM-cre* mice (Fig. 4a, b). Representative hematoxylin and eosin (H&E) images revealed an increase in tumor size and high-grade dysplasia in *LysM-tRXRα* mice (Fig. 4c). Thus tRXRα expression in myeloid cells could promote the growth of AOM/DSS-induced colorectal tumor. The role of tRXRα expression in myeloid cells was also illustrated by increased macrophage infiltration (Fig. 4d) and mRNA expression of IL-6, IL-11, and TNFα (Fig. 4e) in colon tumor tissues from *LysM-tRXRα* but not in *LysM-RXRα* mice. When the activation of STAT3 was examined, we found that it was strongly activated in *LysM-tRXRα* but not in *LysM-RXRα* mice (Fig. 4f), demonstrating that tRXRα expression in myeloid cells is responsible for STAT3 activation. Thus tRXRα expression in myeloid cells can induce IL-6 expression and STAT3 activation in colorectal tumor.

### Activation of the IKK-NF-κB pathway by tRXRα in macrophages

**Fig. 5:**
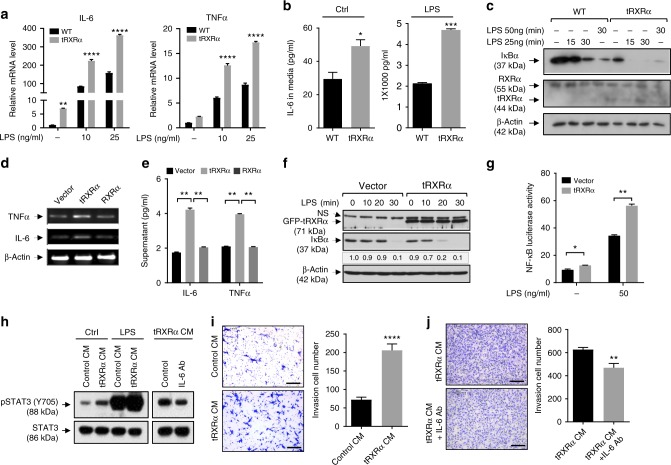
Truncated retinoid X receptor α (tRXRα) activation of the IKK-NF-κB pathway in macrophages. **a** Relative mRNA expression of interleukin (IL)-6 and tumor necrosis factor-α (TNFα) in wild-type (WT) and tRXRα-BMDMs treated with or without lipopolysaccharide (LPS) for 3 h was determined by quantitative reverse transcriptase–PCR (qRT-PCR), two-way analysis of variance (ANOVA). **b** WT and tRXRα-BMDMs cultured with Dulbecco’s modified Eagle’s medium containing 10% fetal bovine serum for 3 days or treated with LPS (25 ng/ml) for 12 h were analyzed by enzyme-linked immunosorbent assay (ELISA), *t* test. **c** Immunoblotting of lysates from WT and tRXRα-BMDMs treated with or without LPS for the indicated time. **d** Expression of IL-6 and TNFα mRNAs in RAW264.7 cells stably expressing green fluorescent protein (GFP), GFP-tRXRα, and GFP-RXRα was analyzed by RT-PCR. **e** Production of IL-6 and TNFα in RAW264.7 cells stably expressing GFP, GFP-tRXRα, and GFP-RXRα was analyzed ELISA, one-way ANOVA. **f** Stable expression of tRXRα in RAW264.7 cells potentiate LPS-induced IκBα degradation determined by immunoblotting. NS nonspecific. **g** Stable expression of tRXRα in RAW264.7 cells promotes LPS-induced NF-κB transactivation determined by reporter assay, two-way ANOVA. **h** Condition medium (CM) collected from WT- and tRXRα-BMDMs cultured for 3 days and treated with or without LPS (25 ng/ml) for 12 h was used to culture mouse CT26 colon cancer cells for 1 h and analyzed for signal transducer and activator of transcription factor 3 activation. Anti-IL-6 antibody (Ab) was incubated with CM of tRXRα-BMDMs for 1 h before being used to culture CT26 cells. **i** CT26 cells were cultured with CM collected from WT- or tRXRα-BMDMs for 24 h in transwell and analyzed by invasion assay. Representative images were photographed and migrated cells per field were counted. Scale bar, 200 μm, *t* test. **j** CM from tRXRα-BMDMs was incubated with or without IL-6 antibody and used to culture CT26 cells for 36 h. Representative images were photographed and migrated cells per field were counted. Scale bar, 200 μm, **P < 0.01 by *t* test. Data are mean ± SEM, *P < 0.05, **P < 0.01, ***P < 0.001, ****P < 0.0001. For immunoblotting, one of three or four similar experiments is shown

To determine the molecular mechanism by which tRXRα expression in inflammatory cells promotes the growth of colorectal tumor through the IL-6-STAT3 pathway, we prepared bone marrow-derived macrophages (BMDMs) from *Tg-tRXRα* mice (tRXRα-BMDMs) and the corresponding control wild-type mice (Supplementary Fig. 4a). tRXRα-BMDMs showed enhanced mRNA expression of IL-6 and TNFα (Fig. 5a) and secretion of IL-6 (Fig. 5b) in the absence or presence of lipopolysaccharide (LPS) when compared to control BMDMs, consistent with data obtained with *LysM-tRXRα* mice (Fig. 4e). As IL-6 is a nuclear factor (NF)-κB-regulated cytokine in inflammatory cells^61,62^, we determined whether tRXRα could promote the activation of the IκB kinase (IKK)-NF-κB inflammatory pathway in macrophages. Treatment of control BMDMs with LPS resulted in IκBα degradation, an indicative of IKK activation, in a time- and dose-dependent manner (Fig. 5c). When tRXRα-BMDMs were analyzed, we found that the basal level of IκBα was much lower in tRXRα-BMDMs as compared to that in control BMDMs, suggesting a role of tRXRα in activating IKK in an LPS-independent manner. tRXRα could also potentiate the effect of LPS, as LPS-induced IκBα degradation occurred much faster in tRXRα-BMDMs than in control BMDMs. The role of tRXRα in activating the IKK-mediated inflammatory pathway was also supported by our data showing that stable expression of tRXRα in RAW264.7 cells (Supplementary Fig. 4b, c) enhanced the mRNA expression of IL-6 and TNFα (Fig. 5d) and their secretion in medium (Fig. 5e), and the effect of LPS on inducing IκBα degradation (Fig. 5f) and NF-κB transactivation (Fig. 5g). Conversely, transfection of RXRα small interfering RNA (siRNA) in THP-1 cells, which inhibited the expression of both RXRα and tRXRα, attenuated the effect of LPS on inducing IκBα degradation (Supplementary Fig. 4d) and activating the NF-κB reporter gene transcription (Supplementary Fig. 4e). LPS-induced mRNA expression of IL-6 and TNFα was also inhibited by RXRα siRNA transfection (Supplementary Fig. 4f). Collectively, these results demonstrated that tRXRα is involved in regulating the IKK-NF-κB pathway in macrophages.

We next studied whether tRXRα activation of the IKK-NF-κB inflammatory pathway in macrophages could serve to activate STAT3 in colon cancer cells. Thus condition medium (CM) from tRXRα-BMDMs were prepared and used to culture mouse CT26 colon cancer cells. Analysis of STAT3 activation in CT26 cells revealed that CM from tRXRα-BMDMs was much more effective than control BMDMs in activating STAT3 (Fig. 5h). Cell invasion assays showed that the invasion of CT26 cells was significantly increased when they were cultured with CM from tRXRα-BMDMs but not from control BMDMs (Fig. 5i). CM from tRXRα-BMDMs could also potentiate the effect of LPS on activating STAT3. Similar results were obtained when THP-1 cells were analyzed. In this case, transfection of tRXRα but not of RXRα strongly induced the expression of IL-6 and TNFα (Supplementary Fig. 5a). CM from tRXRα-transfected but not from RXRα-transfected THP-1 cells enhanced STAT3 activation in human HCT-116 colon cancer cells (Supplementary Fig. 5b). In agreement with enhanced STAT3 activation, cell invasion assays showed that the invasion of colon cancer cells was significantly increased when they were cultured with CM from tRXRα-BMDMs (Fig. 5i) or co-cultured with THP-1 cells expressing tRXRα but not RXRα (Supplementary Fig. 5c). Interestingly, tRXRα-expressing THP-1 cells could also promote the expression of c-Myc and cyclin D1 and growth and invasion of breast cancer cells (Supplementary Fig. 5d–f), indicating that the effect of tRXRα in macrophages is not limited to colon cancer. To determine whether IL-6 secreted from tRXRα-BMDMs played a role in STAT3 activation, CM from tRXRα-BMDMs was incubated with anti-IL-6 antibody. Neutralization of IL-6 activity by anti-IL-6 antibody diminished its effect on inducing STAT3 activation in CT26 cells (Fig. 5h) and their invasion activity (Fig. 5j), revealing a role of IL-6 in STAT3 activation and colon cancer cell growth.

### tRXRα interacts with TRAF6 in macrophages

**Fig. 6:**
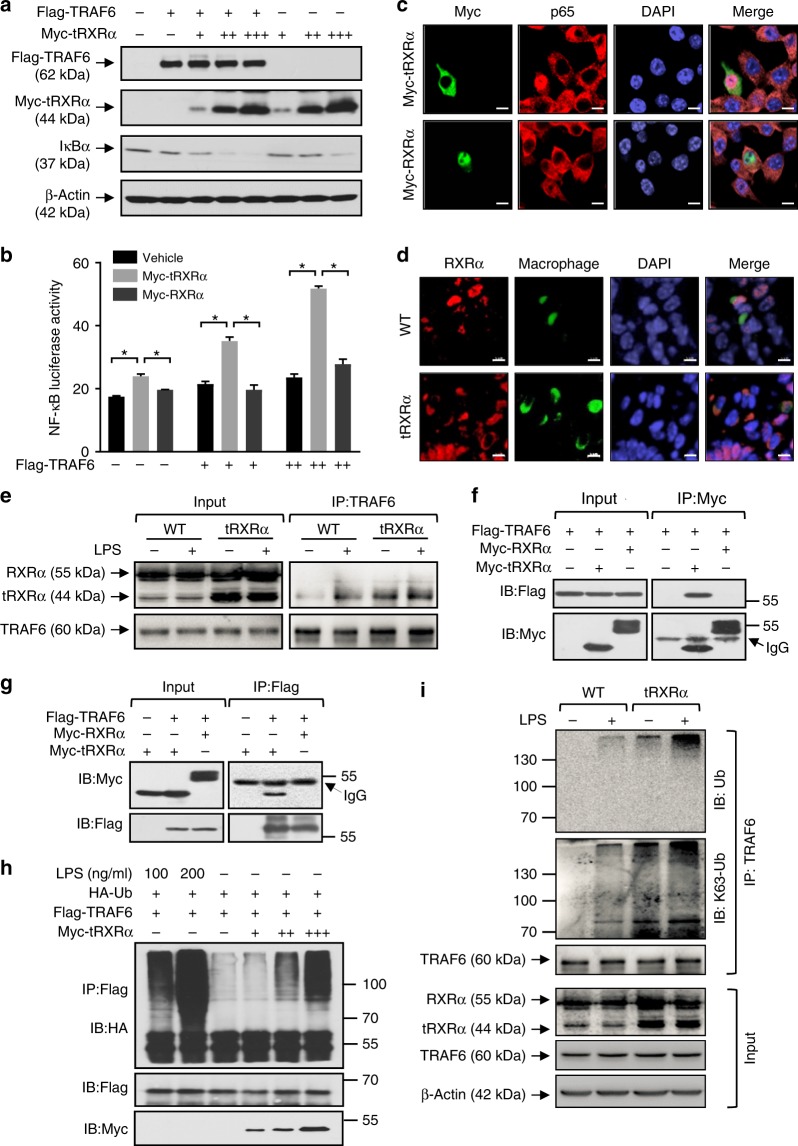
Truncated retinoid X receptor α (tRXRα) interacts with TRAF6 and activates the IKK-NF-κB pathway. **a** Lysates from HEK293T cells transfected with Flag-TRAF6 and/or increasing amounts of Myc-tRXRα were analyzed by immunoblotting. **b** CV-1 cells transfected with pNF-κB reporter alone or in combination with RXRα or TRAF6 plasmids were analyzed for nuclear factor (NF)-κB transactivation by reporter assay. Data are mean ± SEM, *P < 0.05, by two-way analysis of variance. **c** Subcellular localization of transfected Myc-tRXRα or Myc-RXRα in RAW264.7 cell was revealed by immunostaining using anti-Myc antibody. The localization of endogenous p65 was stained by anti-p65 antibody. Green, Myc; red, p65; blue, DAPI. Scale bar, 10 μm. **d** Subcellular localization of RXRα in macrophages of colon tumor tissues from mice treated with azoxymethane/dextran sodium sulfate for 120 days was determined by immunostaining using ΔN197 anti-RXRα antibody. Green, CD68; Red, ΔN197; Blue, DAPI. Scale bar, 5 μm. **e** Wild-type (WT)- and tRXRα-BMDMs treated with lipopolysaccharide (LPS; 50 ng/ml) for 15 min were analyzed for tRXRα interaction with TRAF6 by immunoprecipitation assays using anti-TRAF6 antibody. Immunoprecipitates were analyzed by immunoblotting. **f**, **g** HEK293T cells transfected with the indicated plasmids were analyzed by co-immunoprecipitation assays using anti-Myc or anti-Flag antibody. **h** RAW264.7 transfected with HA-Ub, Flag-TRAF6, and Myc-tRXRα plasmids were immunoprecipitated with anti-Flag antibody and analyzed for TRAF6 ubiquitination by immunoblotting. **i** WT- and tRXRα-BMDMs treated with or without LPS (50 ng/ml) for 15 min were immunoprecipitated with anti-TRAF6 antibody and analyzed for TRAF6 ubiquitination by immunoblotting. For immunoblotting, one of three or four similar experiments is shown

TRAF6, an essential upstream regulator of the IKK complex, is one of the key signaling proteins in the IKK/NF-κB pathway in macrophages^64,65^. TRAF6 is highly expressed in many different types of cancer including colorectal cancer^66,67^. Interestingly, the levels of TRAF6 were significantly elevated in *Tg-tRXRα* mice challenged with either AOM/DSS (Fig. 2a) or DSS (Fig. 2b). Colon tumor from *LysM-tRXRα* mice also showed a significant elevated level of TRAF6 expression, which positively correlated with STAT3 activation (Fig. 4f). These observations promoted us to study whether and how TRAF6 mediated the effect of tRXRα on activating the NF-κB-IL-6-STAT3 cascade. Indeed, cotransfection of TRAF6 augmented the effect of tRXRα on inducing IκBα degradation (Fig. 6a) and NF-κB transactivation (Fig. 6b), suggesting a role of TRAF6 in mediating tRXRα activity.

TRAF6 activation of the IKK complex occurs in the cytoplasm^64,65^. As the first step to study whether tRXRα interacted with TRAF6, we examined the subcellular localization of tRXRα in RAW246.7 cells and found that transfected Myc-tRXRα resided in the cytoplasm while transfected Myc-RXRα was in the nucleus (Fig. 6c). Immunostaining also revealed predominant cytoplasmic RXRα staining in colon tumor from *Tg-tRXRα* mice (Fig. 6d). Thus tRXRα is a cytoplasmic protein in macrophage. We then tested whether tRXRα could interact with TRAF6 in the cytoplasm to modulate IKK activation. Immunoprecipitation of TRAF6 from tRXRα-BMDMs resulted in co-immunoprecipitation of tRXRα, which was slightly enhanced when cells were treated with LPS (Fig. 6e). The interaction appears to be very stable as trace amount of tRXRα expressed in control BMDMs also interacted with TRAF6 in an LPS-dependent manner. The LPS-dependent interaction between endogenous TRAF6 and tRXRα was also observed in THP-1 and RAW264.7 cells (Supplementary Fig. 6a). The interaction between TRAF6 and tRXRα was confirmed by data showing that transfected Myc-tRXRα but not Myc-RXRα interacted with transfected Flag-TRAF6 (Fig. 6f, g). Our mutagenesis analysis revealed that the interaction involved the TRAF domain of TRAF6 and the LBD of tRXRα (Supplementary Fig. 6b–d).

TRAF6 has E3 ligase activity, which can induce autoubiquitination necessary for its activation of IKK^64^. We thus determined whether tRXRα interaction with TRAF6 could result in TRAF6 autoubiquitination. Consistent with previous results^68^, transfected Flag-TRAF6 was strongly ubiquitinated by LPS. Similar to the effect of LPS, transfection of tRXRα promoted TRAF6 ubiquitination in a dose-dependent manner (Fig. 6h). In contrast, transfection of RXRα had no such an effect (Supplementary Fig. 6e). Stable expression of tRXRα in RAW264.7 cells (Supplementary Fig. 6f) or tRXRα expression in BMDMs (Fig. 6i) could potentiate the effect of LPS on inducing TRAF6 ubiquitination. Taken together, these results demonstrated that tRXRα can activate the IKK-NF-κB pathway in macrophages by inducing TRAF6 autoubiquitination through their direct protein–protein interaction.

**Fig. 7:**
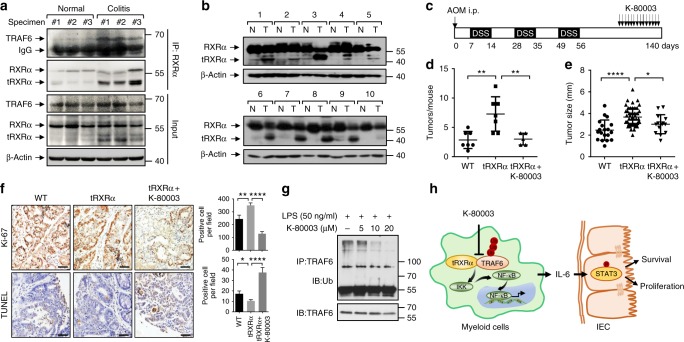
Expression of truncated retinoid X receptor α (tRXRα) in human specimens and the inhibitory effect of K-80003 on tRXRα-mediated colorectal carcinogenesis. **a** tRXRα expression and interaction with TRAF6 in ulcerative colitis patients. Lysates of colorectal tissues from healthy and ulcerative colitis patients were analyzed by immunoblotting and co-immunoprecipitation assay using ΔN197 anti-RXRα antibody. **b** The expression of RXRα/tRXRα were immunoblotting in tumor tissue (T) and corresponding adjacent normal tissue (N) from 10 colorectal tumor patients. **c** Schematic representation of the azoxymethane (AOM)/dextran sodium sulfate (DSS) protocol and K-80003 treatment. Mice were treated with AOM/DSS for 125 days and then administered with vehicle control or K-80003 (20 mg/kg) via oral gavage for 2 weeks daily. **d** Average tumor number. Data points represent the tumor number of one individual mouse. The horizontal bar represents the mean ± SD (*n* = 7/5), one-way analysis of variance (ANOVA). **e** Average tumor size. Each dot represents a tumor. The horizontal bar represents the mean ± SD, one-way ANOVA. **f** Representative images of colon cancer stained with Ki-67 antibody or with the terminal deoxinucleotidyl transferase-mediated dUTP-fluorescein nick end labeling (TUNEL) Kit. Ki67-positive or TUNEL-positive cells per field were counted. Data are mean ± SEM, one-way ANOVA. Scale bar, 100 μm. **g** THP-1 cells pretreated with the indicated concentrations of K-80003 before exposed to lipopolysaccharide (50 ng/ml) were subjected to immunoprecipitation assay using anti-TRAF6 antibody and analyzed for TRAF6 ubiquitination by immunoblotting. **h** Schematic of tRXRα activation of the nuclear factor-κB pathway in macrophages and its role in promoting colon cancer cell survival and proliferation through induction of signal transducer and activator of transcription factor 3 activation. *P < 0.05, **P < 0.01, ***P < 0.001. For immunoblotting, one of three or four similar experiments is shown

To study whether tRXRα production and interaction with TRAF6 is clinic significant, we examined the expression of tRXRα in tissue specimens from patients with ulcerative colitis and colorectal cancer. Our results demonstrated that tRXRα was expressed in colorectal tissues from three ulcerative colitis patients while tRXRα was barely detected in colorectal tissues from three healthy individuals (Fig. 7a). In addition, various degrees of tRXRα expression were found in tumor tissues but not the corresponding adjacent normal tissues from ten colon cancer patients examined (Fig. 7b). Moreover, tRXRα expressed in ulcerative colitis patients could interact with TRAF6. Thus tRXRα production and interaction with TRAF6 appears to have role in the pathogenesis of CAC.

### Inhibition of the tumorigenic effect of tRXRα by K-80003

K-80003 is a potent inhibitor of tRXRα through its unique tRXRα-binding mechanism^52^. Administration of *Tg-tRXRα* mice with K-80003 during the late stage of AOM/DSS-induced colorectal carcinogenesis (Fig. 7c) significantly reduced colon tumor load, reflected by both smaller tumor sizes and less tumor numbers as compared to vehicle-treated mice (Fig. 7d, e; Supplementary Fig. 7a, b). The potent anti-CAC effect of K-80003 was associated with its inhibition of cell proliferation and induction of apoptosis (Fig. 7f), suppression of the mRNA expression of IL-6, TNFα, and IL-11 and the serum production of IL-6, and inhibition of STAT3 activation (Supplementary Fig. 7c–e). These data demonstrated that K-80003 is a potent inhibitor of tRXRα-mediated IL-6-STAT3 signaling during colorectal tumorigenesis. In vitro, K-80003 inhibited LPS induction of mRNA expression of IL-6 (Supplementary Fig. 7f) in a dose-dependent manner in macrophages. It also suppressed the effect of LPS on inducing IκBα degradation, NF-κB transactivation, and nuclear translocation (Supplementary Fig. 7g–i) in RAW264.7 cells. Mechanistically, K-80003 inhibited LPS-induced interaction of tRXRα with TRAF6 (Supplementary Fig. 7j) and TRAF6 ubiquitination (Fig. 7g). Together, these results demonstrated that K-80003 is a potent inhibitor of tRXRα-mediated CAC development by preventing tRXRα interaction with TRAF6.

## Discussion

Our characterization of the *tRXRα* transgenic mice revealed a critical role of tRXRα in the development of CAC. *Tg-tRXRα* mice displayed increased tumor frequency and enlarged tumor size when applied to the AOM/DSS protocol (Fig. 1). Colorectal tumors developed from *Tg-tRXRα* mice showed much more profound macrophage infiltration and expressed high levels of inflammatory cytokines (Fig. 3), demonstrating that the tumorigenic effect of tRXRα is due to its modulation of the tumor inflammatory microenvironment. This was supported by our discovery that tRXRα expression in myeloid cells could promote colorectal carcinogenesis (Fig. 4) and is in agreement with previous reports that RXRα plays an important role in regulating macrophage functions and inflammatory disorders^33,35^. The role of tRXRα in myeloid cells was further illustrated by our in vitro experiments showing that ectopic expression of tRXRα but not of RXRα in macrophages potentiated LPS induction of IκBα degradation, NF-κB nuclear translocation, and expression of several cytokines (Fig. 5). Thus the tumorigenic role of tRXRα during CAC development is largely attributed to its activation of the IKK-NF-κB pathway in macrophages. Our observations that tRXRα is produced in colorectal tissues of ulcerative colitis patients (Fig. 7a) and a majority of tumor tissues but not the corresponding adjacent normal tissues from patients with colorectal tumor (Fig. 7b), suggest the clinical relevance of tRXRα expression in the development of CAC.

Recent studies have provided important insight into the molecular mechanisms linking chronic inflammation to the development of cancers. Of particular important mechanism is the activation of STAT3 by IL-6^61,62^. However, the regulation of the IL-6-STAT3 pathway during the development of CAC is still obscure, which has hindered the development of effective strategies targeting the pathway. Our present data demonstrated that tRXRα is an important modulator of the IL-6-STAT3 cascade during colorectal tumorigenesis. Enhanced STAT3 phosphorylation was observed in *Tg-tRXRα* mice before (Fig. 2a) or after treatment with AOM/DSS (Fig. 2a) or DSS alone (Fig. 2b). Induction of STAT3 phosphorylation by tRXRα was fast, occurring in 1 day after DSS treatment (Fig. 2f), suggesting a pivotal role of tRXRα in the activation of STAT3. TNFα, another cytokine known to activate the NF-κB pathway, whose activation contributes to tumor development, was also upregulated in *tRXRα* mice (Fig. 3). TNFα activation of the NF-κB signaling induces the expression of inflammatory mediators and growth factors. We previously reported that tRXRα could promote TNFα activation of the PI3K/AKT signaling pathway, another important pathway critical for cancer cell proliferation and survival^25^. AKT activation was indeed enhanced in tRXRα transgenic mice challenged with AOM/DSS (Fig. 2a) or DSS (Fig. 2b). Taken together, these results identified tRXRα as an important factor that mediates the causal link between chronic inflammation and the development of CAC.

While how RXRα binds to DNA and regulates target gene transcription is well understood, the non-transcriptional actions regulated by RXRα and the underlying mechanisms remain poorly understood. Given the fact that transcription-independent action of RXRα and other nuclear receptors often involves their interaction with important signal transduction proteins critical for the growth and death of cancer cells, understanding the molecular mechanism by which nuclear receptors exert their non-transcriptional action may offer new strategies to explore their therapeutic potential. One of the unique properties of tRXRα is its cytoplasmic localization under inflammatory conditions^9^. Our investigation of the molecular mechanism by which tRXRα activates the inflammatory pathway in macrophages revealed a previously unrecognized non-transcriptional role of tRXRα through its interaction with TRAF6. TRAF6 is overexpressed in a variety of cancers including colon cancer^66,67^. TRAF6 overexpression in NIH3T3 cells resulted in NF-κB activation, anchorage-independent growth, and tumor formation, whereas TRAF6 depletion inhibited cancer cell proliferation and tumorigenesis^69^, demonstrating its oncogenic activity. As TRAF6 plays a key role in the assembly of protein complexes necessary for the activation of the IKK-NF-κB pathway, TRAF6 overexpression would lead to constitutive NF-κB activation. Indeed, we found that TRAF6 expression was enhanced in *Tg-tRXRα* mice in the absence or presence of AOM/DSS treatment (Fig. 2a). Moreover, TRAF6 expression was increased when tRXRα was expressed in myeloid cells (Fig. 4f). While how tRXRα induces TRAF6 expression in myeloid cells is currently unknown, we showed that coexpression of TRAF6 and tRXRα synergistically induced IκBα degradation (Fig. 6a) and NF-κB activation (Fig. 6b). Such an effect was likely due to their physical interaction (Fig. 6), which led to autoubiquitination of TRAF6, an event known to activate the IKK/NF-κB pathway^64^. Interestingly, TRAF6 interacted with tRXRα but not with RXRα, demonstrating that tRXRα activation of the IKK-NF-κB pathway is a tumor-specific event. Thus tRXRα activation of the NF-κB pathway in macrophages through its interaction with cytoplasmic TRAF6 represents an important non-transcriptional mechanism by which tRXRα promotes tumor growth.

Our discovery that tRXRα plays a pivotal role in modulating the IL-6-STAT3 pathway offers an effective and selective approach to treat inflammation-associated cancers. NSAIDs have demonstrated promising preventive and therapeutic effects against colorectal cancer. Sulindac, one of the most studied NSAIDs, is effective against colorectal cancer in various clinical studies^53,54^. Unfortunately, long-term sulindac treatment has several drawbacks, such as gastrointestinal bleeding associated with COX1 inhibition and increased cardiovascular risk resulting from COX2 inhibition^70^. Our previous discovery that tRXRα serves as an intracellular target of sulindac led to our identification of K-80003, a sulindac analog with enhanced tRXRα binding and diminished COX inhibition activities^25^. We showed here that K-80003 is a potent inhibitor of tRXRα-mediated colorectal carcinogenesis by inhibiting tRXRα-mediated NF-κB-IL-6-STAT3 pathway (Fig. 7). The effect of K-80003 is likely due to its ability to induce tRXRα tetramerization^52^ that may serve to inhibit tRXRα interaction with TRAF6. It is noteworthy that K-80003 exhibits very desirable pharmacological profiles including much reduced side effects in animals^25^, which could be attributed to its effective and selective induction of tetramerization of tRXRα but not of RXRα^52^. The selective effect of K-80003 is expected to lead to specific blockade of tRXRα in tumor cells without compromising the normal function of RXRα. The ability of K-80003 to bind to distinct and alternate hydrophobic grooves on the surface of tRXRα^52^ may also contribute to its efficacy and selectivity, as tRXRα may employ its surface grooves to interact with non-transcriptional signaling proteins such as TRAF6.

Together, our results revealed an oncogenic effect of tRXRα in the development and growth of CAC through its activation of the IL-6-STAT3 signaling pathway and uncovered a new tRXRα non-transcriptional mechanism responsible for the activation. Our data also identify K-80003 as a promising anti-CAC agent that targets the tRXRα pathway.

## Methods

### Mice

*LoxP-tRXRα* and *loxP-RXRα* mice were constructed by using pronuclear microinjection. The detail sequences are presented in Supplementary Fig. 1a and the sequence of *loxp-tRXRα* are the same with *loxp-RXRα* except that 85 N-terminal amino acids in RXRα were deleted in *loxp-tRXRα*. *CMV-Cre* mice (B6.C-Tg(*CMV-cre*)*1Cgn/J*) (Cat.J006054) and *LysM-cre* mice (B6.129P2-*Lyz2tm1(cre*)Ifo/NJU) (Cat.J004781) were kindly provided by Professor Jia-huai Han (Xiamen University). All of the mice were approved by the Animal Care and Use Committee of Xiamen University and maintained with 12-h light/12-h dark cycles at the Laboratory Animal Center in Xiamen University.

### Antibody and reagent

Anti-RXRα (D20) (Cat. sc-553,1:1000), anti-RXRα (ΔN197) (Cat. sc-774,1:1000), anti-Myc (9E10) (Cat. Sc-40, 1:1000), anti-TRAF6 (H-274) (Cat. sc-7221, 1:1000), anti-AKT (Cat. sc-8312, 1:1000), anti-β-catenin (E-5, 1:1000) (Cat. sc-7963), anti-PCNA (FC-261, 1:100 dilution for immunohistochemistry) (Cat. sc7907), and anti-p65 (Cat. sc-109) antibodies were from Santa Cruz Biotechnology (Santa Cruz, CA, USA). Anti-IκBα (Cat. ab32518, 1:1000), anti-pSTAT3 (Y705) (Cat. ab76315, 1:100,000), anti-Ki-67 (Cat.ab15580, 1:200 dilution for immunohistochemistry), and anti-CD68 (Cat. ab31630, 1:400 dilution for immunohistochemistry) antibodies were from Abcam (UK). Anti-Ubiquitin (Cat. 3933S), anti-STAT3 (Cat. 9139S, 1:1000), anti-p-IκBα (Cat. 9246, 1:1000), anti-K63-ubiquitin (D7A11) (Cat.#5621, 1:1000), anti-TRAF6 (D21G3) (Cat. 8028S, 1:1000), anti-pGSK3β (Ser9) (Cat.#9336, 1:1000), and anti-pAKT (S473) (Cat. 9271S, 1:1000) antibodies were from Cell Signal Technology (Beverly, MA, USA). Anti-GSK3β (total) (Cat.610202, 1:1000) was from BD biosciences (San Diego, USA). Anti-β-actin (Cat. A5441, 1:50,000) and anti-FLAG (Cat. F1804, 1:1000) antibodies were from Sigma (St. Louis, MO, USA). DSS (Cat. 0216011080100g) (MW:36000-50000) was from MP Biomedicals. AOM (Cat. A5486), LPS (Cat. L2630), RXRα siRNA (SASI_Hs01_00097639, SASI_Hs01_00097640, SASI_Rn02_00260246), and nonspecific control siRNA were from Sigma. TianScript RT Kit (Cat. KR104-02) was from TianGen Biotechnology (Beijing, China). Mouse IL-6 (Cat. KE10007) and TNFα (Cat. KE10002) enzyme-linked immunosorbent assay kits for IL-6 and TNFα were from Proteintech (Wuhan, China).

### Induction of colitis and CAC

Mice (8–12-week old, male) were injected intraperitoneally with a single dose of AOM (10 mg/kg; Sigma, #A5486). After 7 days, 2.5% DSS (MP Biomedicals, #0216011080100g) was given in the drinking water for 7 days, followed by 14 days of regular drinking water. The DSS treatment was repeated for two additional cycles, and mice were sacrificed 120 days after the AOM injection. In the K-80003 treatment model, K-80003 (dissolved in dimethyl sulfoxide (DMSO) and diluted with 0.9% NaCl containing 5.0% (V/V) Tween-80, 20 mg/kg) were administered via oral gavage daily in last 2 weeks. Normal saline with DMSO and 5.0% Tween-80 was employed as the vehicle control. For short-term colitis and inflammation studies, mice (8–10-week old) were given 3% DSS for 5 days, followed by regular drinking water for 4 days and sacrificed at the indicated time points. Body weights were recorded daily. Colons were removed from mice, flushed with cold phosphate-buffered saline (PBS), opened longitudinally, fixed as “swiss-rolls” in 4% formalin solution (Sigma, #16005-1KG-R), and paraffin embedded. Before fixing the colons, tumor size measurements were performed using a vernier caliper in a blinded fashion.

### Clinical tumor samples

Human colorectal cancerous and para-cancerous tissues were obtained from Zhongshan Hospital, Xiamen University, and human colorectal tissues from normal and ulcerative colitis patients were obtained from Soochow University hospital with patient informed consent and approval of the Medical Ethical Committee of Zhongshan Hospital and Soochow University hospital, respectively.

### Histopathological analysis and immunohistochemistry

Paraffin-embedded colon sections were stained with H&E. The extent of inflammation was measured and scored. Paraffin-embedded slides were deparaffinized. Antigen unmasking was carried out by heating in microwave in 10 mM sodium citrate buffer for 5 min and cooling for 2 min, with repeat of this process five times. Slides were incubated with primary antibodies in PBS. Horseradish peroxidase secondary anti-rat or anti-rabbit antibodies (BIOTnA, cat#TAHC03D) were added and incubated at room temperature for 1 h. The sections were stained with DAB substrate and counterstained with hematoxylin. 10–15 fields were randomly selected from each section at high magnification, photographed, and analyzed by two pathologists previously uninformed. Histological scores were assigned by experimenters “blinded” to sample identity. Colonic epithelial damage was assigned scores as follows: 0 = normal; 1 = hyperproliferation, irregular crypts, and goblet cell loss; 2 = mild-to-moderate crypt loss (10–50%); 3 = severe crypt loss (50–90%); 4 = complete crypt loss, surface epithelium intact; 5 = small-to-medium-sized ulcer (<10 crypt widths); 6 = large ulcer (≥10 crypt widths).

### Real-time polymerase chain reaction

Total RNAs were extracted by Trizol (Invitrogen) and complemental DNA were synthesized using TIANScript RT Kit (Tiangen). Real-time PCR was performed using SYBR Green Master Mix following the manufacturer’s instructions (Roche) on ABI Stepone PCR instrument using respective primers (Supplementary Table 1). Expression data were normalized to GAPDH mRNA expression.

### Cell culture and transfection

RAW264.7, CV-1 cells, THP-1, and HEK293T were purchased from the American Type Culture Collection (Manassas, VA, USA). CT26.WT and HCT-116 were purchased form Cell Bank in Chinese Academy of Sciences in Shanghai. RAW264.7, CV-1 cells, and HEK293T cells were cultured in Dulbecco’s modified Eagle’s medium (DMEM) with 10% fetal bovine serum (FBS); THP-1 cells were maintained in RPMI 1640 containing 10% fetal bovine serum in a humidified atmosphere containing 5% CO_2_ at 37 °C. They were regularly tested for mycoplasma contamination using the MycoFluor^TM^ Mycoplasma Detection Kit (ThermoFisher Scientific) and always found to be negative. Transient transfection of HEK293T cells were carried out with calcium phosphate method. RAW264.7 cells, THP-1 cells, and CV-1 cells were transfected with Lipofectamin 2000 (Invitrogen). RAW264.7 stable cells lines expressing GFP, GFP-RXRα, and GFP-tRXRα were selected with 600 μg/ml G418 for 3–4 weeks.

### Isolation and culture of BMDMs

Bone marrow cells were flushed from the femurs and tibias and treated with red blood cell lysis buffer (BD Biosciences, cat555899) to get rid of blood cell. Single-cell suspensions were obtained by forcing through a 40-mm cell strainer and differentiated into macrophages in DMEM supplemented with 10% FBS, 30% L929 CM, and 1% penicillin and streptomycin for 6–7days. Primary BMDMs were cultured with DMEM supplemented with 10% FBS for experiments.

### Condition medium and colony-formation assay

BMDMs were cultured with DMEM supplemented with 10% FBS for 72 h. THP-1 cells were cultured with RPMI1640 without FBS for 48 h. These media were collected, centrifuged with 10,000 × *g* for 20 min and stored at −80 °C. For culturing tumor cells, CM were warmed up at 4 °C and used to culture tumor cells. In the colony assay, media were used to culture tumor cells for 2 weeks in 6-well plate and colonies were fixed with methylalcohol and stained with Giemsa.

### Cell invasion assay

Cell invasion assay was performed with an invasion chamber (BD Biosciences, cat #354480) pretreated with medium 2 h for matrix membrane hydration. Tumor cells (HCT116 or MDA-MB-231, 2.5 × 10^4^ per well, 1% FBS) were seeded in the upper chamber, and the inflammatory cells (THP-1 cell, 1 × 10^5^ per well, 1% FBS) were loaded into the lower chamber. After 36 h of incubation at 37 °C in 5% CO_2_, noninvasive cells were removed from the upper surface of the transwell membrane with a cotton swab, and the invaded cells on the lower membrane surface were fixed with paraformaldehyde (PFA) and stained with Gimasa. Randomly selected fields were photographed and the numbers of migrated cells per field were counted. For invasion assay using primary BMDMs and murine CT26 colon cancer cells, CM from BMDMs were placed in the lower chamber and CT26 colon cell were seeded in the upper chamber.

### Plasmid constructions

Expression constructs for TRAF6 was kindly provided by Dr. Ashley Mansell (Monash Institute of Medical Research, Australia). TRAF6/ΔTRAF and TRAF6/ΔRING were generated by PCR and inserted into *pCMV-Myc* vector. Plasmids encoding RXRα, RXRα/1–135, RXRα/1-235, and RXRα/223-462 were constructed using pEGFP-C1 vector.

### Immunoprecipitation and ubiquitination assay

Cells were lysed in a buffer containing 2 mmol/l Tris-HCl (pH7.4), 10 mmol/l EDTA, 100 mmol/l NaCl, and 1% IGEPAL. Whole-cell lysates were subjected to immunoprecipitation with antibodies against RXRα (D20), RXRα (ΔN197), TRAF6, or c-Myc in protein A/G beads (Santa Cruz Biotechnology). For detection of TRAF6 ubiquitination, 10 mM *N*-ethylmaleimide was included in the lysis buffer containing a protease inhibitor “cocktail” (Roche).

### Immunoblotting (IB)

IB was performed as described previously^21,25,52^. Briefly, cell or tissue lysates were electrophoresed by sodium dodecyl sulfate-polyacrylamide gel electrophoresis and transferred to polyvinylidene difluoride membranes. The membranes were blocked with 5% skimmed milk in TBST (50 mM Tris-HCl (pH 7.4), 150 mM NaCl, and 0.1% Tween20) for 1 h, then incubated with primary antibodies and secondary antibodies and detected using enhanced chemiluminescence. The original immunoblots from the main figures are shown in Supplementary Figure 8.

### Confocal microscopy

Cells seeded on glass slides were washed with PBS and fixed in 4% PFA for 15 min. Fixed cells were permeabilized with PBS containing 0.1% Triton X-100 for 15 min and blocked with 1 mg/ml bovine serum albumin in PBS for 30 min at room temperature. Primary antibodies were incubated for 3 h at room temperature or overnight at 4 °C and secondary antibodies were incubated at room temperature for 1 h. DAPI was dyed for visualizing nuclei. The images were taken under an LSM-510 confocal laser scanning microscope system (Carl Zeiss).

### Luciferease report assay

RAW264.7 and THP-1 cells were transfected with plasmids combined with pNF-κB-Luc for NF-κB reporter. After transfection for 24 h (48 h for siRNA), cells were lysed by reporter lysis buffer. Firefly and Renilla luciferase activities were analyzed with the Dual-Luciferase Reporter Assay system (Promega). Transfection and expression efficiency were normalized to Renilla luciferase activity

### Statistical analysis

Data are presented as mean ± standard error of the mean (SEM) or ±standard deviation (SD), and statistical significance are reported in the figure legends. Statistics was analyzed using Student’s *t* test, chi-square test, or analysis of variance (ANOVA) analysis (one-way ANOVA for comparisons between groups, two-way ANOVA for comparisons the magnitude of changes between different groups from different cell lines). *P* < 0.05 was considered statistically significant (*), *P* < 0.01 as highly significant (**), *P* < 0.001 as extremely significant (***), and ns as not significant.

### Reporting summary

Further information on experimental design is available in the Nature Research Reporting Summary linked to this article.

## Supplementary information

Supplementary Information

Description of Additional Supplementary Files

Supplementary Data 1

Reporting Summary

## Data Availability

Supporting data of this study are available from the corresponding author on reasonable request. The source underlying Figs. 2a, b, f, 4f, 5c, f, h, 6a, e–i, 7a, b, g and Supplementary Figs. 1c, 4c, 4d, 5a, 5c, 6a, 6c–f, 7e, 7g, 7j are provided as a Supplementary Data 1. A reporting summary for this article is available as a Supplementary Information file.
