# Supplementary material for: Oncogenic potential of truncated RXRα during colitis-associated colorectal tumorigenesis by promoting IL-6-STAT3 signaling
Source: Nat Commun. 2019 Apr 1;10:1463. doi: 10.1038/s41467-019-09375-8 (PMC6443775; doi:10.1038/s41467-019-09375-8)
Supplement: Supplementary file 1 — Supplementary Information [file 41467_2019_9375_MOESM1_ESM.pdf]

Supplementary Information

Oncogenic potential of truncated RXRα during colitis-associated colorectal tumorigenesis by promoting IL-6-STAT3 signaling

Ye et al.

Supplementary Table 1

Supplementary Table 1. Primers used for quantitative RT-PCR.

Gene	Forward primers (5’-3’)	Reverse primers (5’-3’)
**GAPDH**	TTGATGGCAACAATCTCCAC	CGTCCCGTAGACAAAATGGT
**TNFα**	AGGGTCTGGGCCATAGAACT	CCACCACGCTCTTCTGTCTAC
**IL-6**	CTCTGCAAGAGACTTCCATCCAGT	GAAGTAGGGAAGGCCGTGG
**IL-11**	TGTTCTCCTAACCCGATCCCT	CAGGAAGCTGCAAAGATCCCA
**Cyclin D2**	GCGTGCAGAAGGACATCCA	CACTTTTGTTCCTCACAGACCTCTAG
**RegIIIγ**	TGCTGCTCTCCTGCCTGATG	ATAGGAGCCATAGGCACGGG
**Tff3**	TTGCTGGGTCCTCTGGGATAG	TACACTGCTCCGATGTGACAG

Supplementary Figure 1

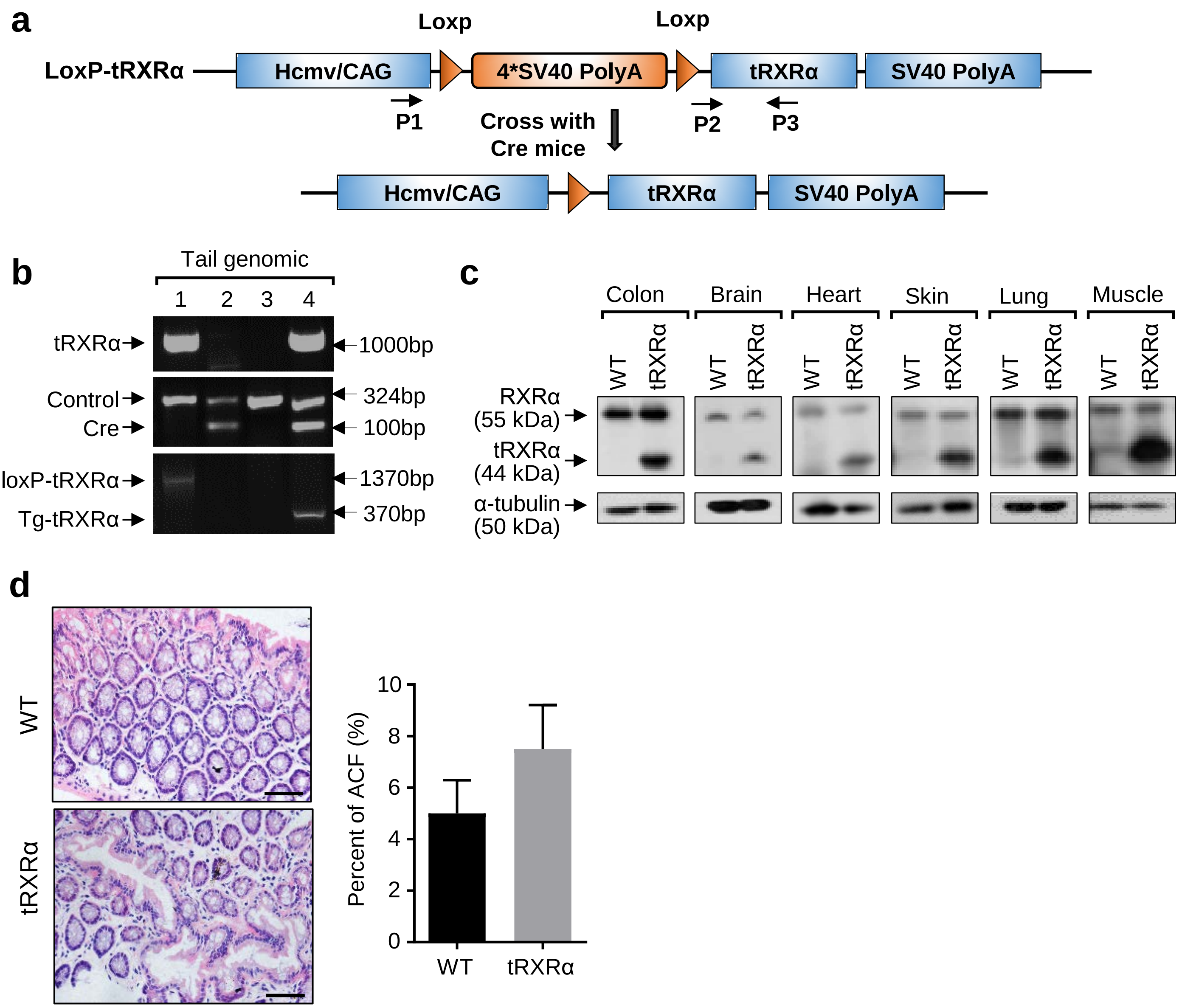

Supplementary Figure 1. Generation and characterization of *Tg-tRXRα* mice.
(**a**) Schematic representation of the production of *Tg-tRXR* mice. Mouse tRXRα cDNA cloned into the pCMV/CAG-loxp/4*SV40/loxp plasmid to generate a transgene vector. Transgenic mice on a C57BL/6 background were generated by pronuclear microinjection, which were crossed with Cre mice to generate Tg-*tRXRα* transgenic mice. P1,P2,and P3 primers were designed to detect loxP-tRXRα and Tg-tRXRα.
(**b**) Tail genomic analysis of Tg-*tRXRα* mouse by PCR. 1, loxP-tRXRα; 2, CMV-cre; 3, wild-type; 4, Tg-tRXRα. The presence of tRXRα sequences in loxP-tRXRα and Tg-tRXRα mice was illustrated by using P2 and P3 primers. By using P1 and P3 primers, the bands of loxP-tRXRα (1370 bp) and Tg-tRXRα (370bp) were detected, demonstrating the deletion of loxp-SV40-loxp sequences from *Tg-tRXRα* mouse.
(**c**) Expression of tRXRα in different tissues from loxP-*tRXRα* (WT) and *Tg-tRXRα* mice was determined by immunoblotting.
(**d**) Representative H&E images of colon sections from *loxP-tRXRα* (WT) and *Tg-tRXRα* mice (21-month old) and examined for microscopic aberrant crypt foci (ACF) after H&E. Data are mean ± SEM (n=6), Scale bar, 50 μm.

Supplementary Figure 2

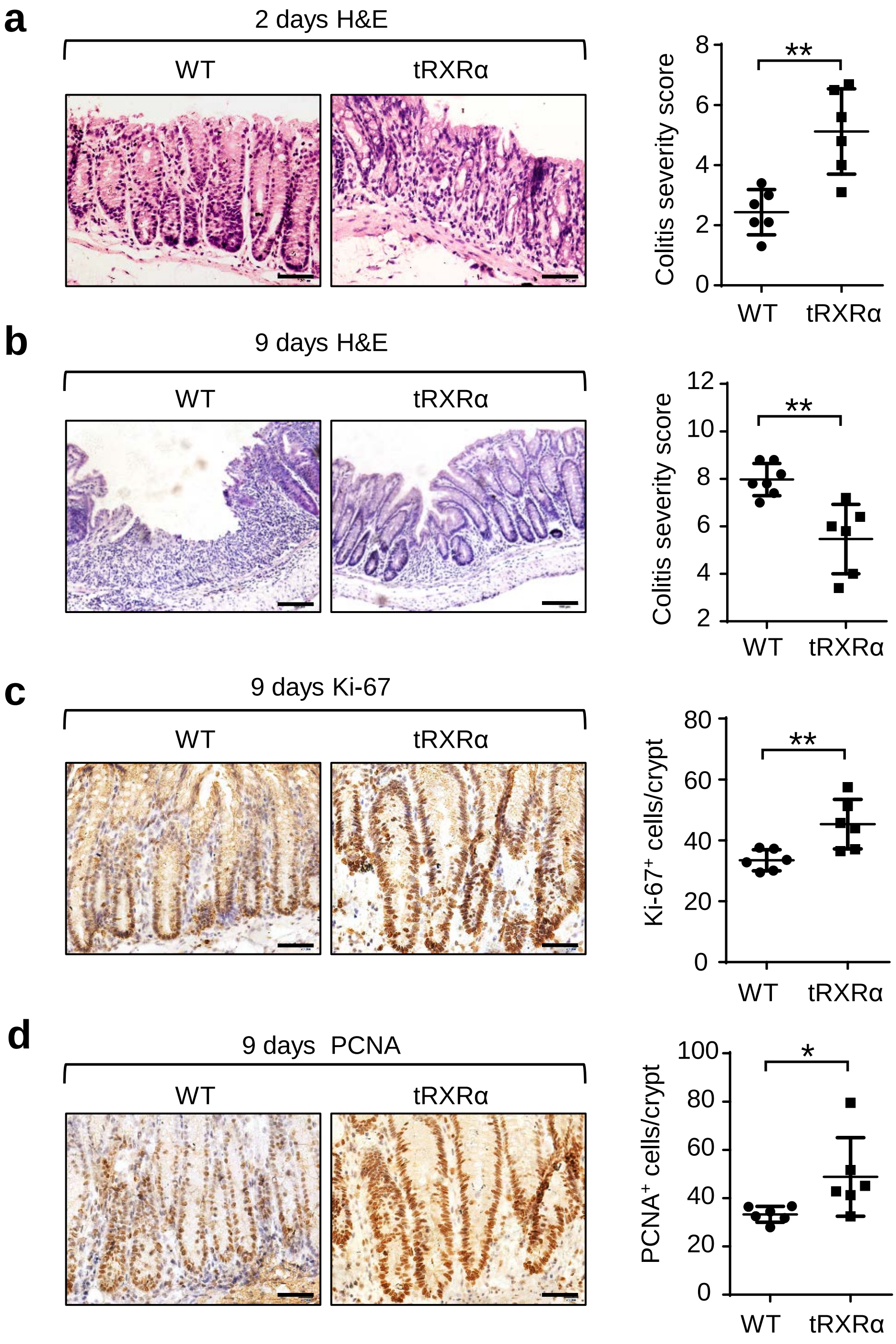

Supplementary Figure 2. tRXRα promotes inflammation and intestinal cell proliferation.

(**a, b**) Representative H&E images of colon sections from mice treated with 3%DSS for 2 days (**a**) or with 3%DSS for 5 days followed by 4 days recovery (**b**). Crypt damage with histopathology was scored. Data are mean ± SD (n=6), ** $p< 0.01$ by t test. Scale bar, 100 µm.

(**c, d**) Representative micrographs of colonic sections from mice administrated with 3%DSS for 5 days followed by 4 days recovery were stained for Ki-67 (**c**) or PCNA (**d**). (scale bar, 100µm) and analyzed The numbers of Ki-67-positive or PCNA-positive cells per crypt were analyzed by counting 18-20 full-length crypts per mouse colon. Data are mean ± SD (n=6). *$p < 0.05$; **$p < 0.01$ by t test. Scale bar, 100µm.

Supplementary Figure 3

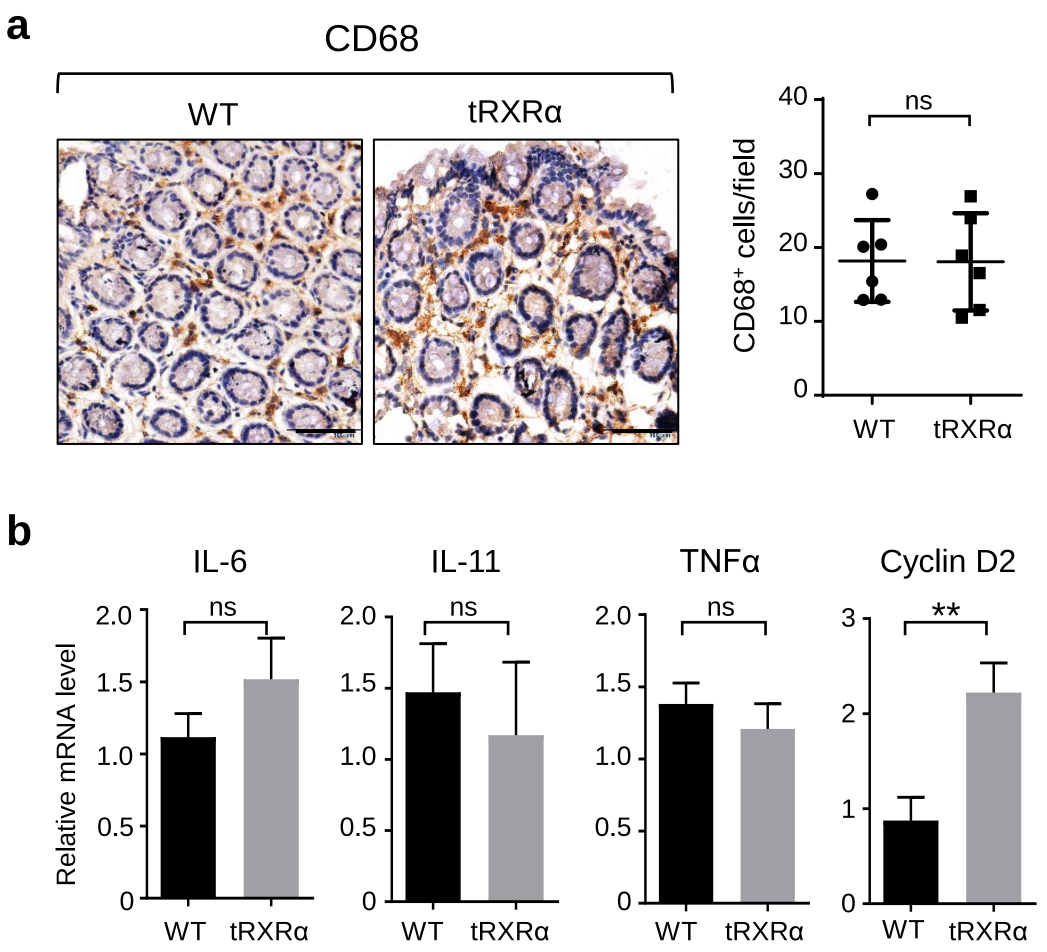

Supplementary Figure 3. tRXRα does not promote inflammation in AOM-induce colorectal carcinogenesis.
(**a**) Representative micrographs of colonic sections from mice treated with the AOM protocol for 6 months were stained for CD68 and analyzed the number of CD68-positive cells per field. Each dot represents a mouse. 10 fields were counted per mice. Data are mean ± SD (n=6); ns, no significant. Scale bar, 100 μm.
(**b**) Relative mRNA expression levels of the indicated genes in colon tissue from mice treated with the AOM protocol for 6 months were determined by qRT-PCR. Data are mean ± SEM (n = 5), ***p* < 0.01 by t test; ns, no significant.

Supplementary Figure 4

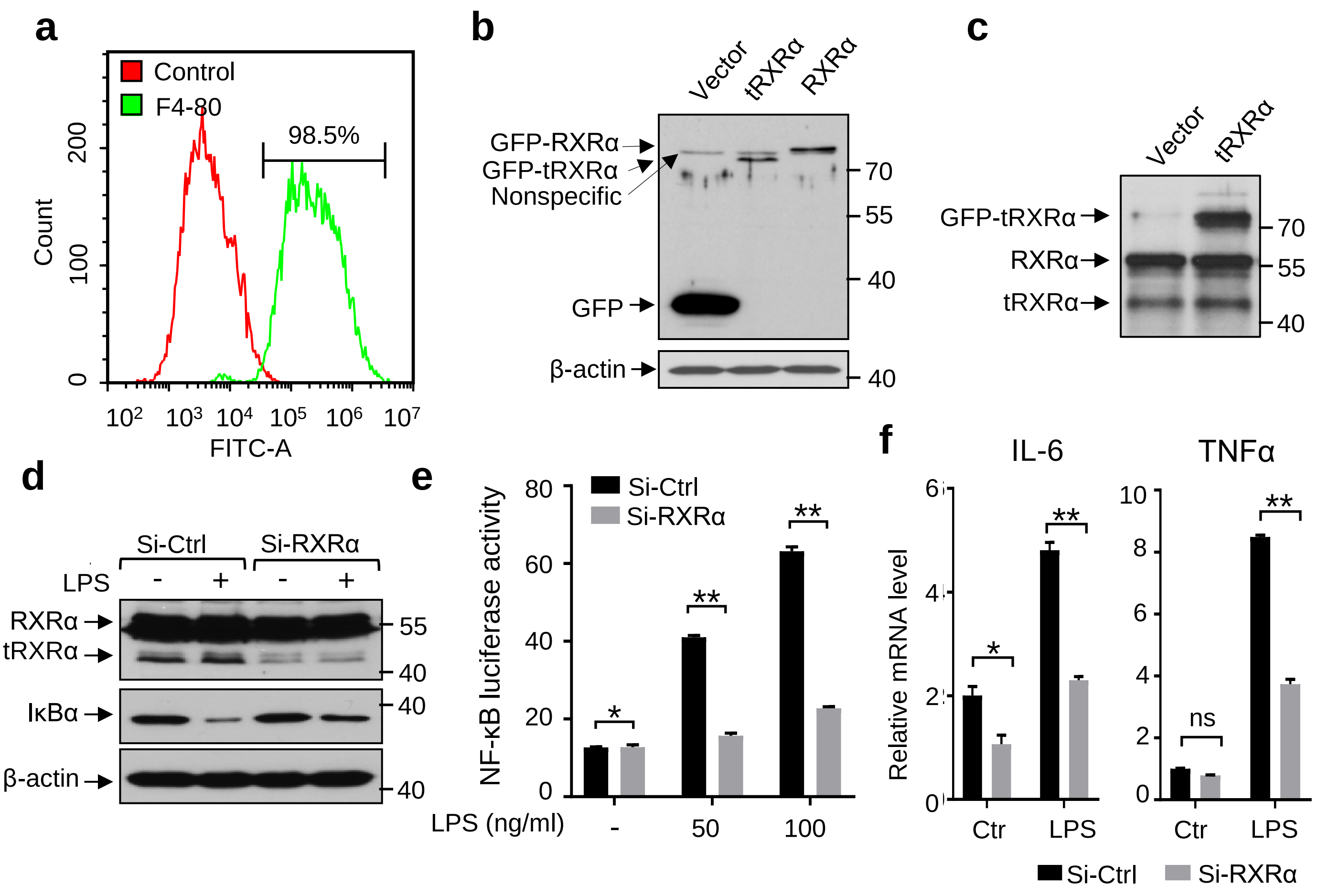

Supplementary Figure 4. tRXRα activation of the IKK-NF-κB pathway in macrophages.
(**a**) Flow cytometric analysis of the purity of BMDMs from bone marrow, which were differentiated and treated with or without macrophages marker F4/80 (green).
(**b,c**) Stable expression of tRXRα and RXRα in RAW264.7 cells. The GFP-tRXRα, GFP-RXRα or GFP Vector plasmid were transfected into RAW264.7 cells, and stable clones were selected with G418 (600 μg/ml) and analyzed by immunoblotting using anti-GFP antibody (**b**) or ΔN197 anti-RXRα antibody in order to compare the expression level of transfected and endogenous tRXRα (**c**).
(**d**) Lysates from THP-1 cells transfected with control or RXRα siRNA were treated with LPS (50 ng/ml) for 30 min and analyzed by immunoblotting.
(**e**) THP-1 cells transfected with control or RXRα siRNA were treated with LPS and analyzed for NF-κB transactivation by reporter assay.
(**f**) Expression of IL-6 and TNFα mRNAs in THP-1 cells transfected with control or RXRα siRNA and treated with or without LPS was analyzed by RT-PCR.
Data are mean ± SEM. *$p < 0.05$, **$p < 0.01$ by two-way ANOVA.

Supplementary Figure 5

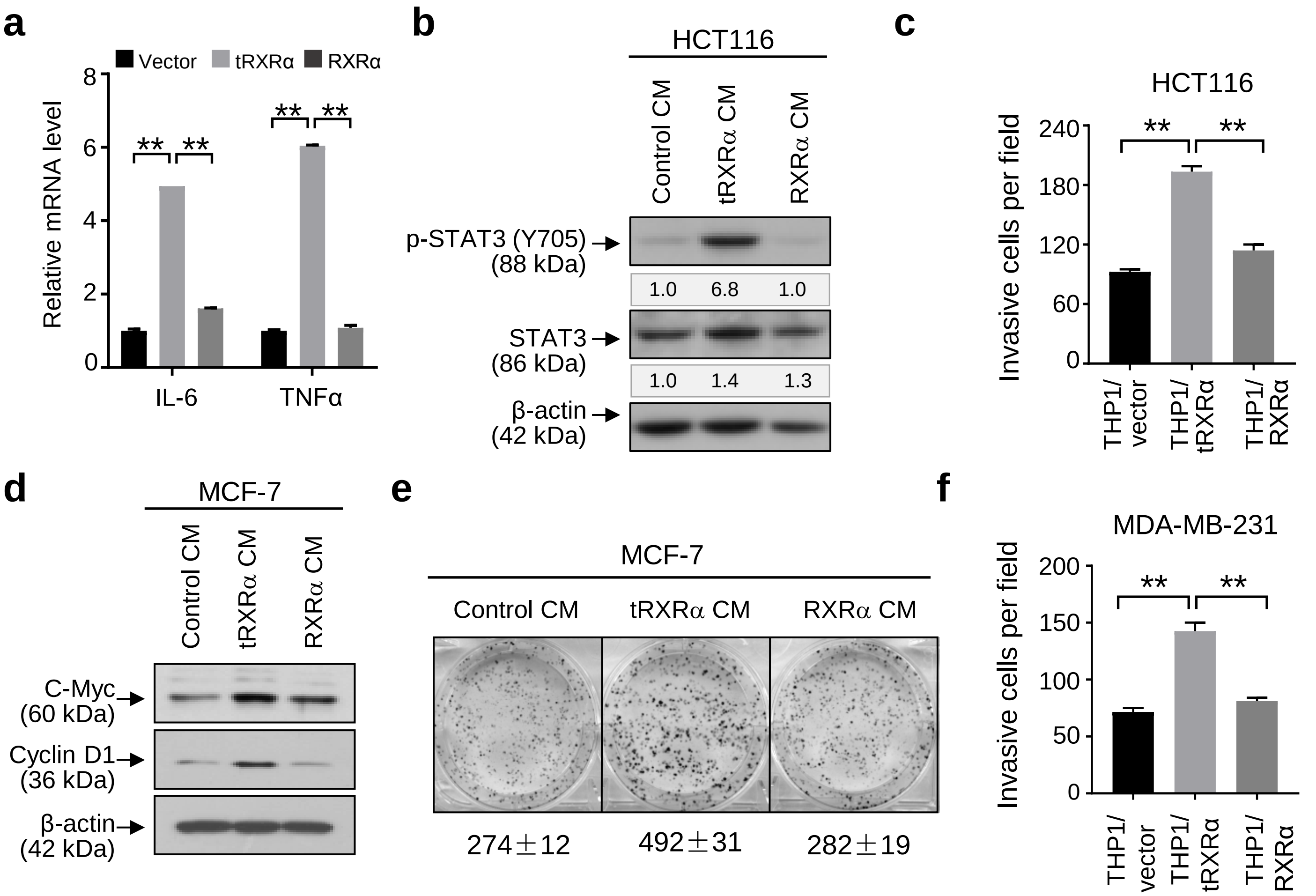

Supplementary Figure 5. tRXRα activation of the IKK–NF-κB pathway in macrophages.

(**a**) Relative mRNA expression of IL-6 and TNFα in THP-1 cells transfected with tRXRα or RXRα were determined by qRT-PCR.

(**b,c**) Effect of CM from THP-1 cells transfected with tRXRα or RXRα on STAT3 activation on HCT116 colon cancer cells and their invasion. The expression of pSTAT3 and STAT3 was determined by immunoblotting (**a**) and the invasive ability of HCT116 cells upon co-culture was determined by invasion assay (**b**).

(**d,e**) Effect of CM from THP-1 cells transfected with tRXRα or RXRα on STAT3 activation of breast cancer cells and their growth. MCF-7 cells cultured with CMs for 24 h were analyzed for gene expression by immunoblotting (**c**). Alternatively, they were cultured with CMs for 2 weeks and analyzed for their colony formation (**d**). The number of colonies was counted per dish.

(**f**) THP-1 cells expressing tRXRα promotes the invasion of MDA-MB-231 breast cancer cells. MDA-MB-231 breast cancer cells were co-cultured with THP-1 cells expressing tRXRα or RXRα, and their invasive ability was determined by in vitro invasion assay. Data are mean ± SEM. ***p* < 0.01 by one-way ANOVA.

Supplementary Figure 6

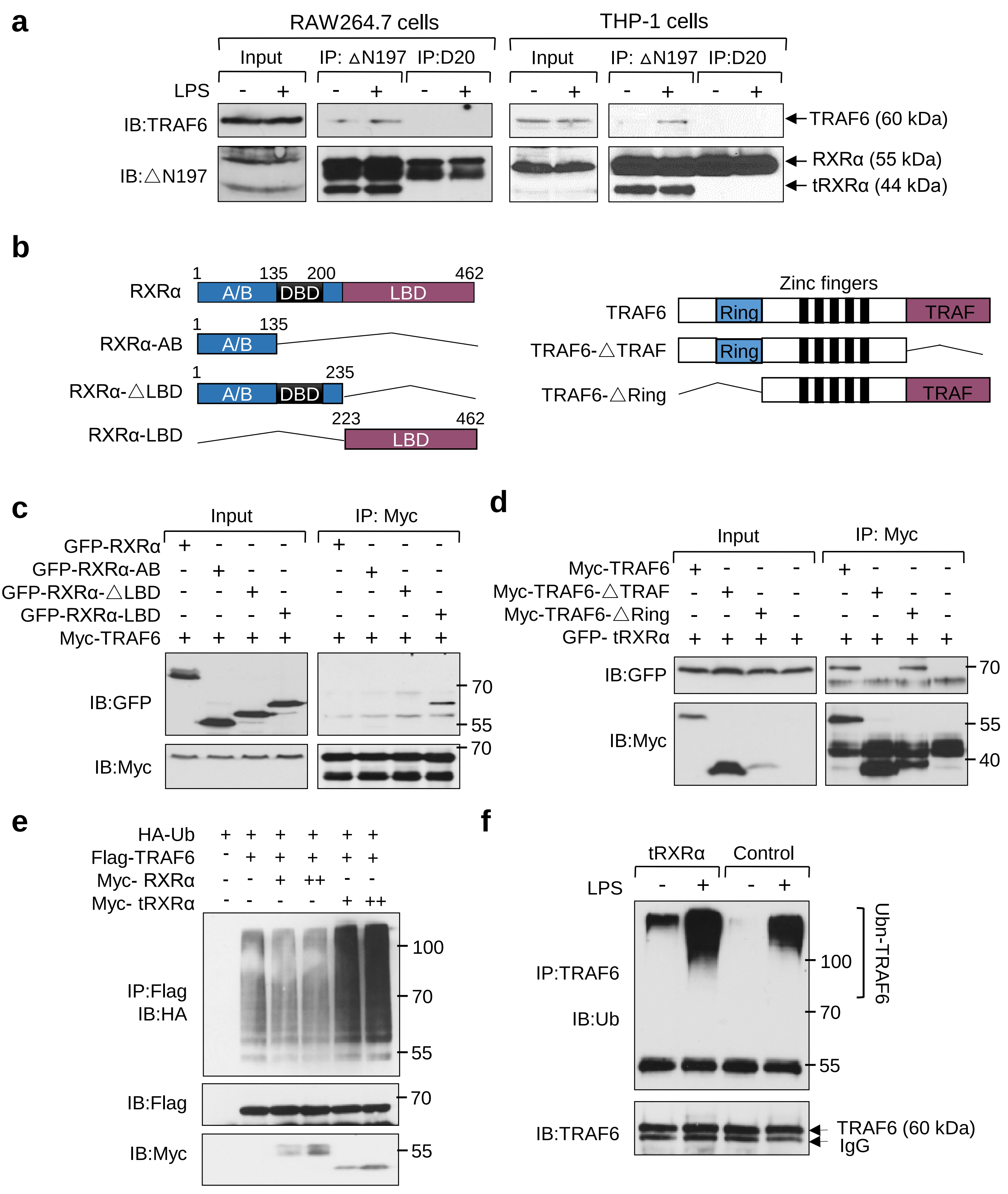

Supplementary Figure 6. Characterization of tRXRα interaction with TRAF6.

(**a**) RAW264.7 cells and THP-1 cells treated with LPS (50 ng/ml) for 20 min were analyzed for tRXRα interaction with TRAF6 by co-immunoprecipitation assays using D20 and ΔN197 anti-RXRα antibodies. Immunoprecipitates were analyzed by immunoblotting (IB).

(**b**) Schematic representations of RXRα and TRAF6 and their mutants.

(**c**) LBD of RXRα is important for its interaction with TRAF6. The mutants of RXRα vectors were transfected into HEK293T cells and analyzed by immunoprecipitation using anti-Myc antibody.

(**d**) TRAF domain of TRAF6 is required for its interaction with tRXRα. The mutants of TRAF6 vectors were transfected into HEK293T cells and analyzed by immunoprecipitation using anti-Myc antibody.

(**e**) tRXRα but not RXRα promoted TRAF6 ubiquitination. HEK293T transfected with HA-Ub, Flag-TRAF6 together with Myc-RXRα or Myc-tRXR plasmids were immunoprecipitated with anti-Flag antibody. Immunoprecipitates were then analyzed for TRAF6 ubiquitination by immunoblotting.

(**f**) RAW264.7 cells stably expressing GFP or GFP-tRXRα were treated with or without LPS (50 ng/ml) for 30 min were immunoprecipitated with anti-TRAF6 antibody and analyzed for TRAF6 ubiquitination by immunoblotting.

Supplementary Figure 7

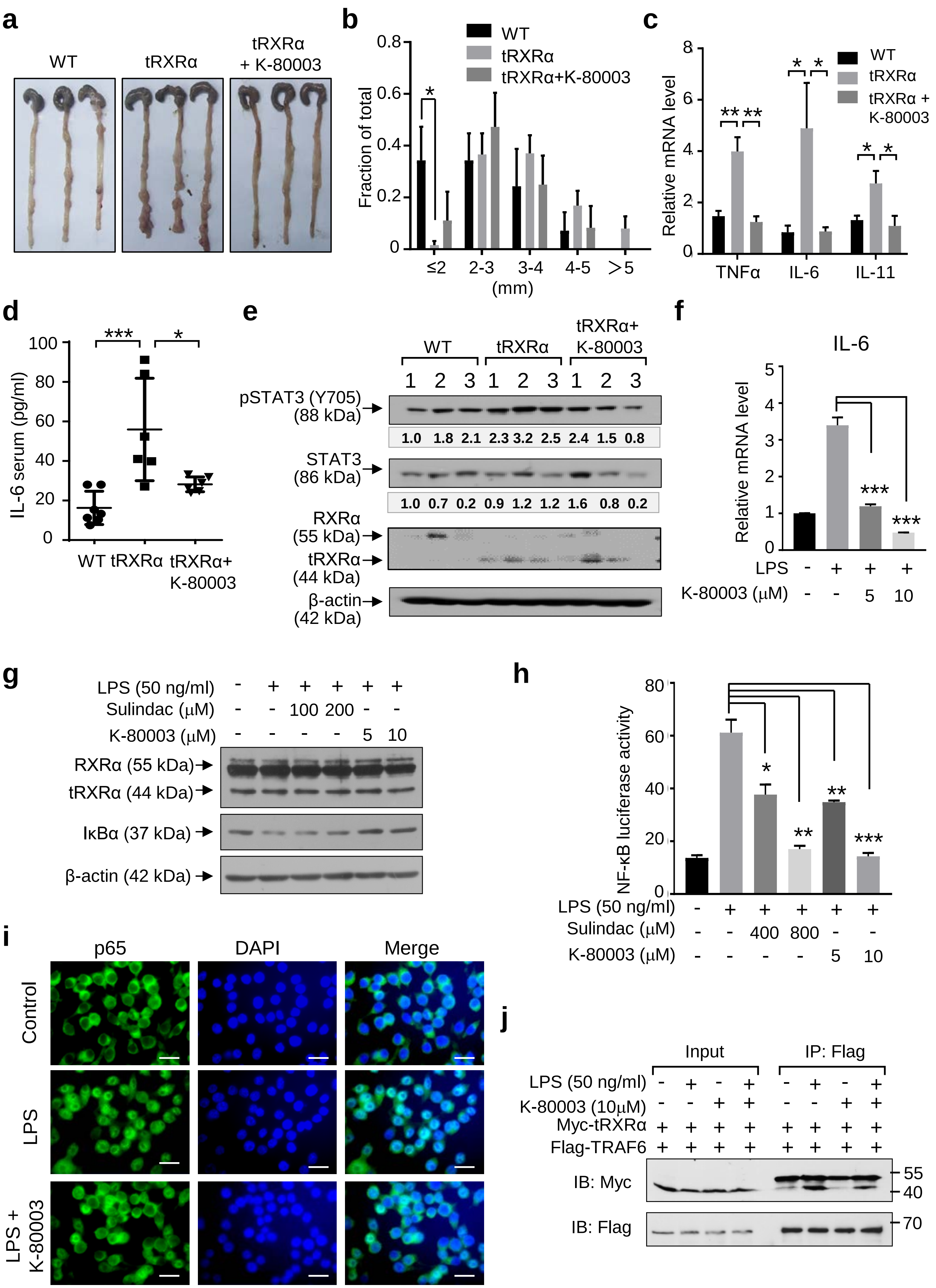

Supplementary Figure 7. Effect of K-80003 on inhibiting tRXRα-dependent development of CAC.

(**a**) Representative images of colon tumors from mice treated with AOM/DSS in the presence or absence of K-80003.

(**b**) Tumor size distribution. Data are mean ± SEM (n = 7/5), by two-way ANOVA.

(**c**) Relative mRNA expression levels of TNFα, IL-6 and IL-11 were determined by qRT-PCR. Data are mean ± SEM (n≥4), by one-way ANOVA.

(**d**) Serum levels of IL6 from mice treated with 3%DSS and/or K-80003 (20 mg/kg) were measured by ELISA. Each dot represents a mouse. . Data are mean ± SD (n = 6), by one-way ANOVA.

(**e**) Colon lysates from mice treated with AOM/DSS and vehicle control or K-80003 were analyzed by immunoblotting. The bands were quantified with image J and normalized to β-actin.

(**f**) Relative mRNA expression of IL-6 in RAW264.7 cells upon treatment with LPS and/or the indicated concentrations of K-80003 for 3 h was analyzed by RT-PCR. Data are mean ± SEM, by one-way ANOVA.

(**g**) RAW264.7 cells treated with the indicated concentrations of K-80003 or Sulindac for 1 hr before exposed to LPS (50 ng/ml) for 30 min were analyzed by immunoblotting.

(**h**) RAW264.7 cells transfected with pNF-κB reporter plasmid were treated with LPS, Sulindac, or K-80003, and analyzed for NF-κB transactivation by reporter assay. Data are mean ± SEM, by one-way ANOVA.

(**i**) RAW264.7 cells pretreated with K-80003 (10 μM) for 1 h before exposed to LPS (50 ng/ml) for 30 min were immunostained with anti-p65 antibody. Scale bar, 10 μm.

(**j**) RAW264.7 cells transfected with Flag-TRAF6 and Myc-tRXRα were treated with K-80003 (10 μM) for 1 h before exposed to LPS (50 ng/ml) for 30 min, and their interaction was analyzed by co-immunoprecipitation assay.

*$P<0.05$, ** $P<0.01$, ***$p<0.001$.

Fig. 2a

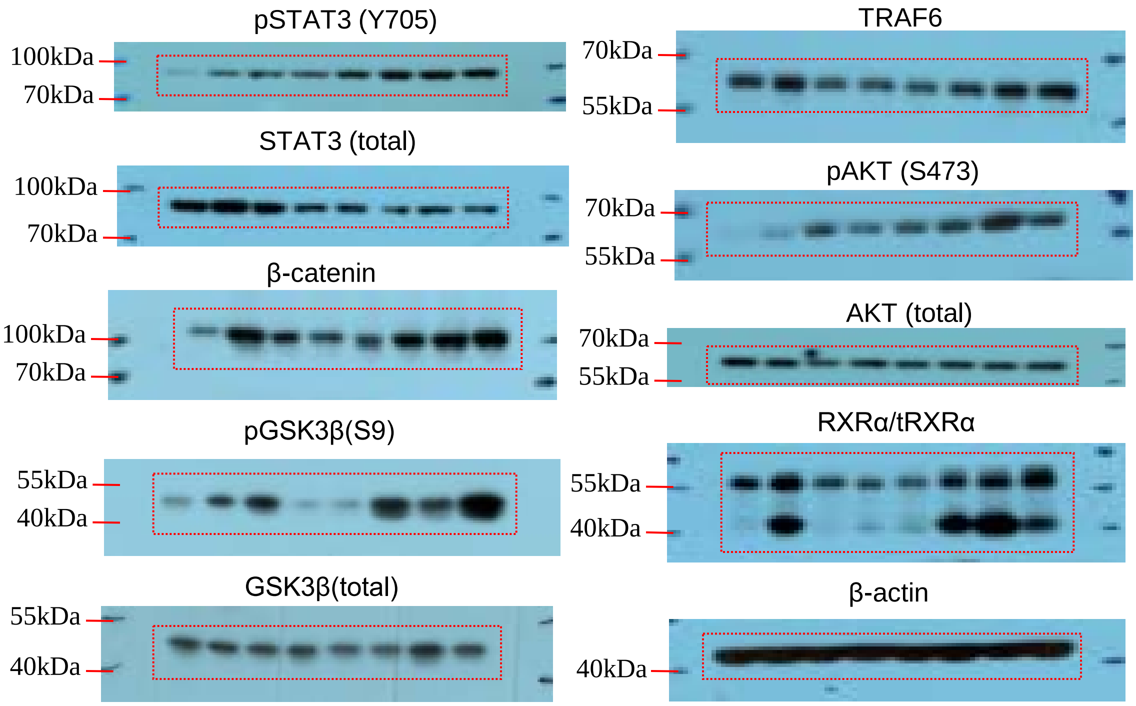

Fig. 2b

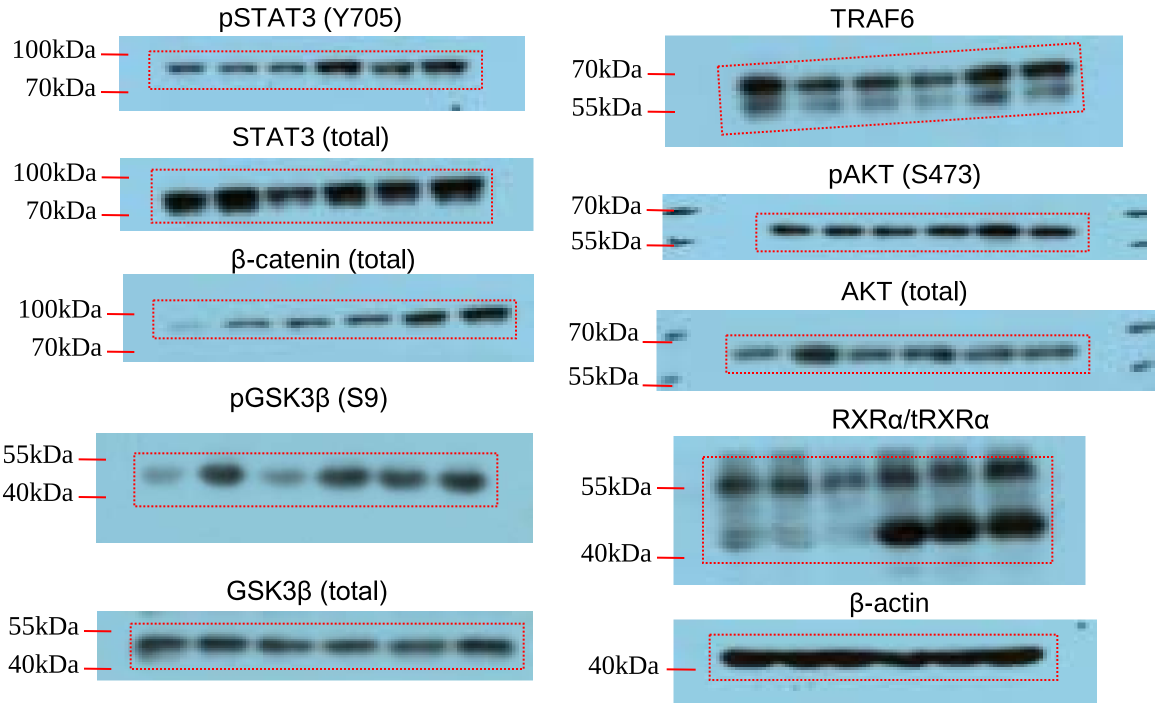

Fig. 2f

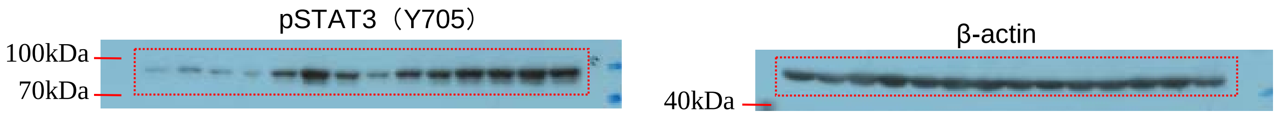

Fig. 4f

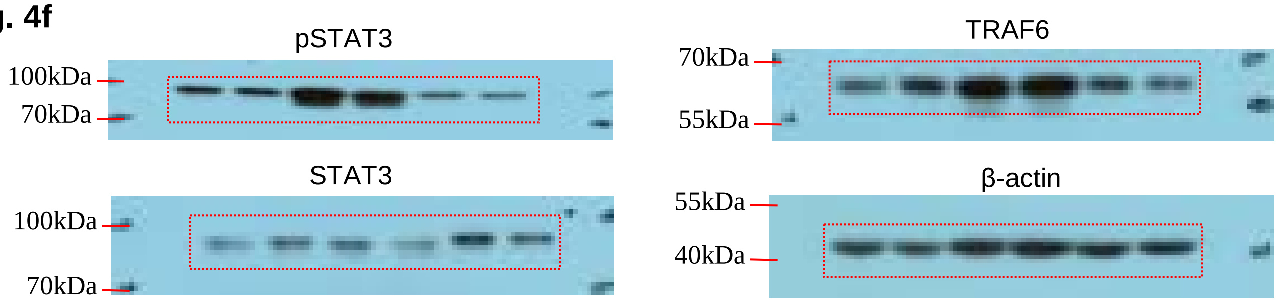

Fig. 5c

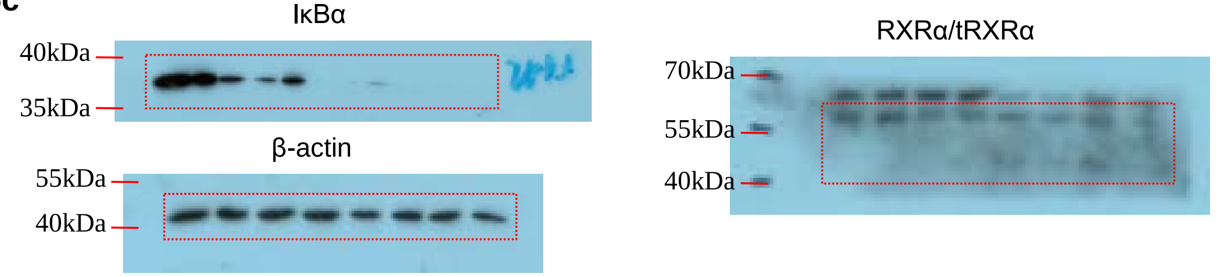

Fig. 5f

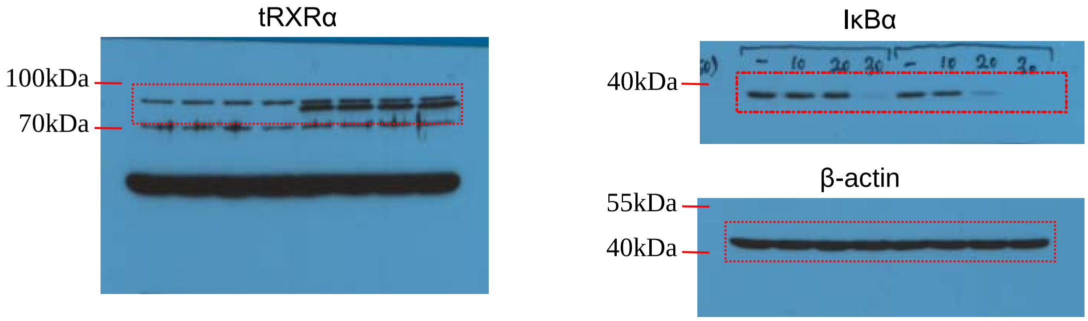

Fig. 5h

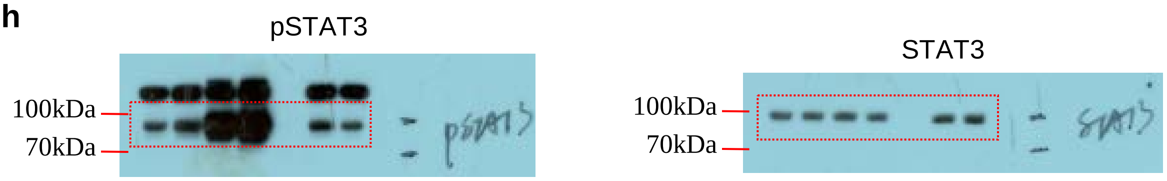

Fig. 6a

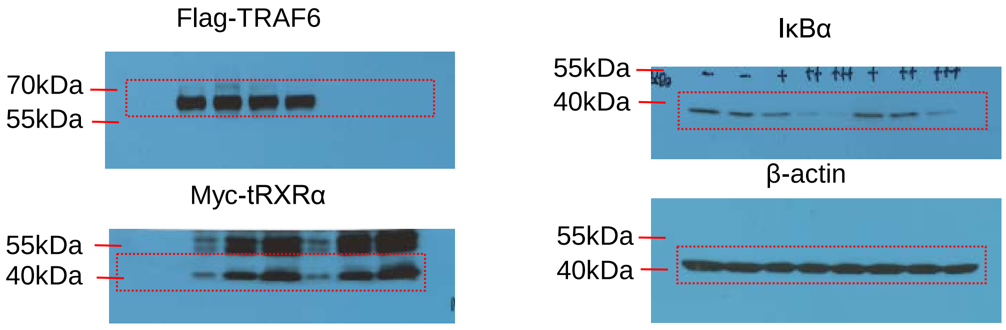

Fig. 6e

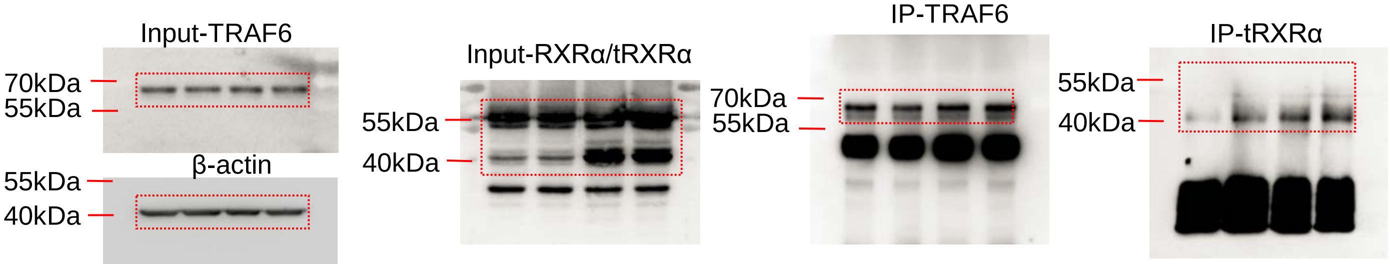

Fig. 6f

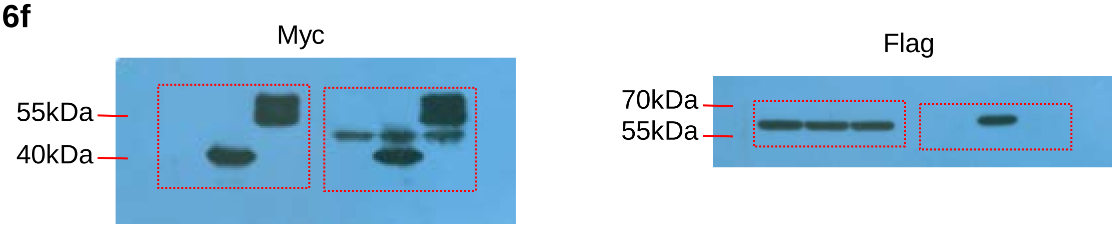

Fig. 6g

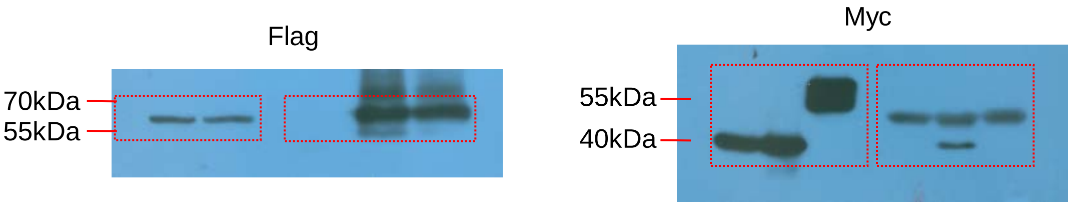

Fig. 6h

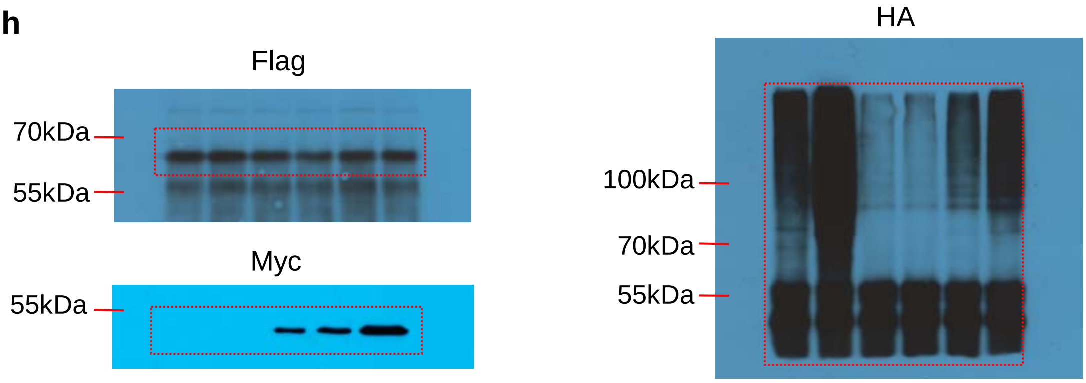

Fig. 6i

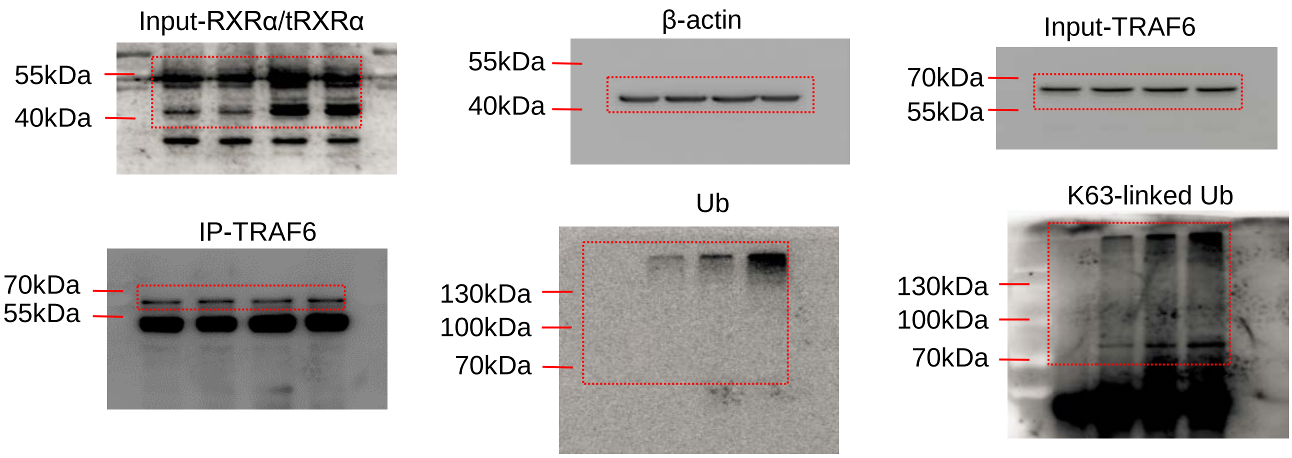

Fig. 7a

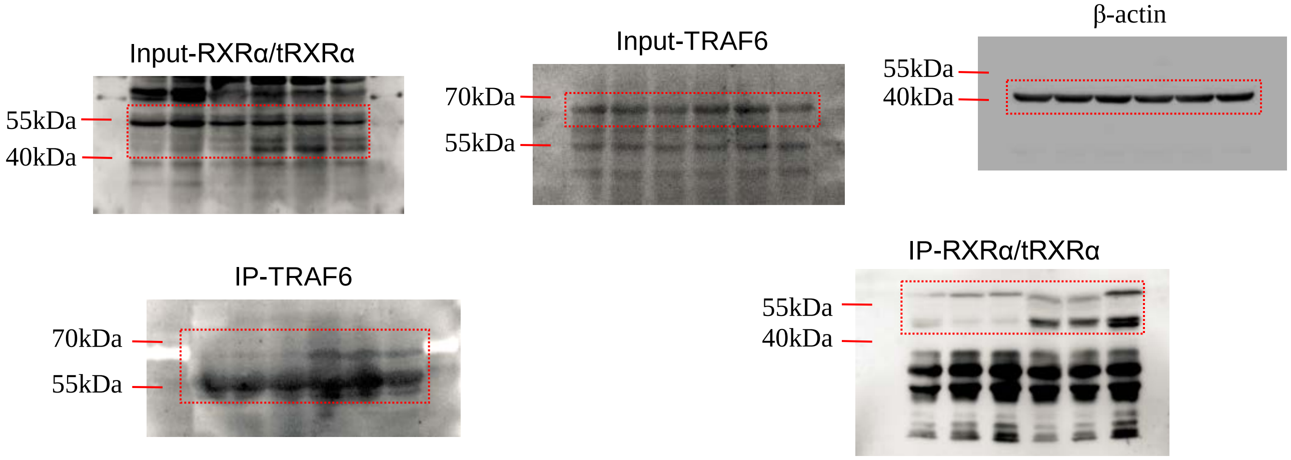

Fig. 7b

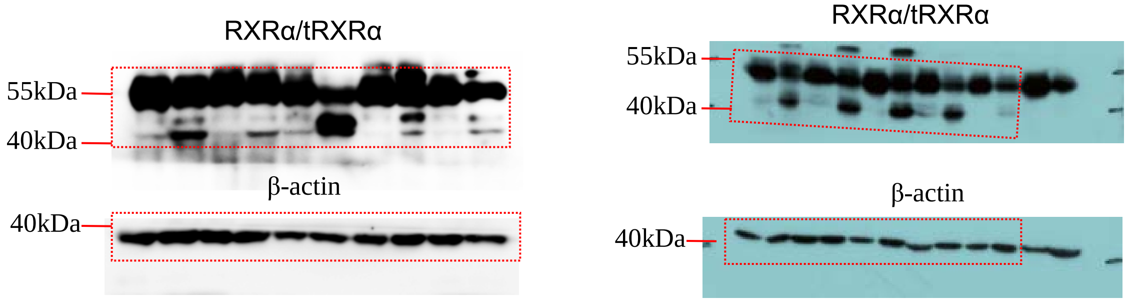

Fig. 7g

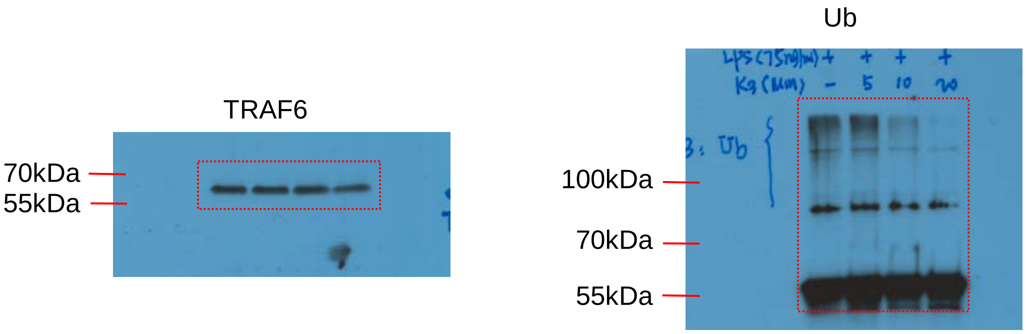

Fig. S1c

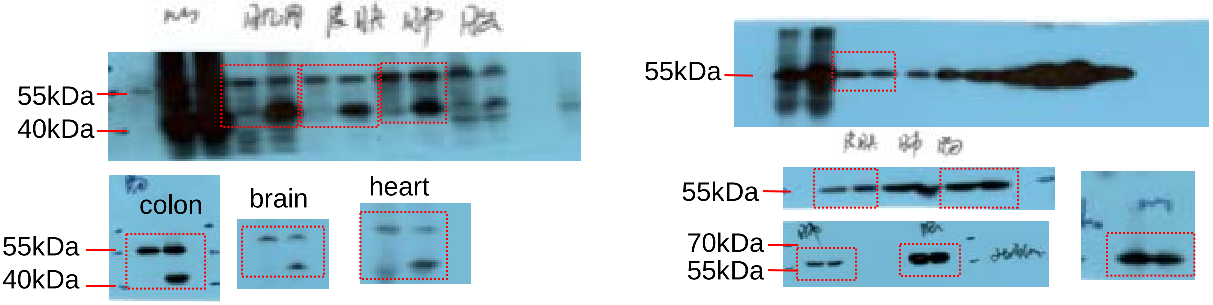

Fig. S4c

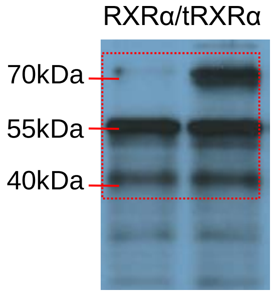

Fig. S4d

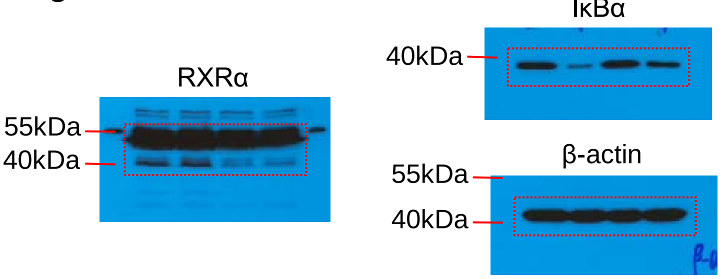

Fig. S5a

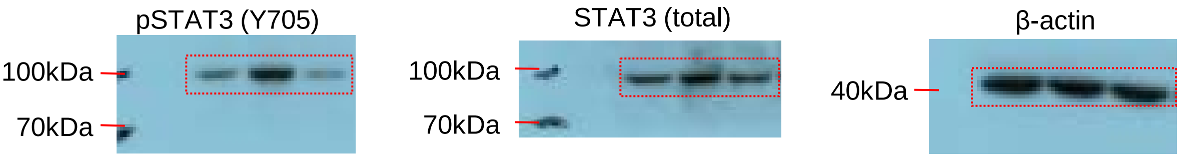

Fig. S5c

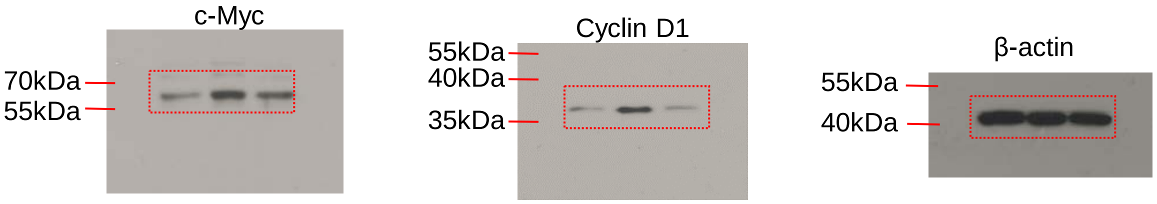

Fig. S6a

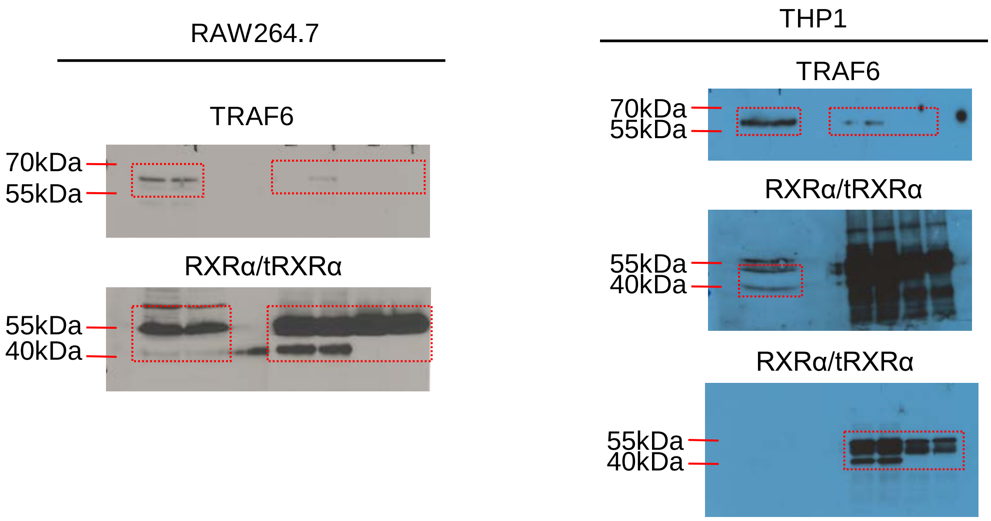

Fig. S6c

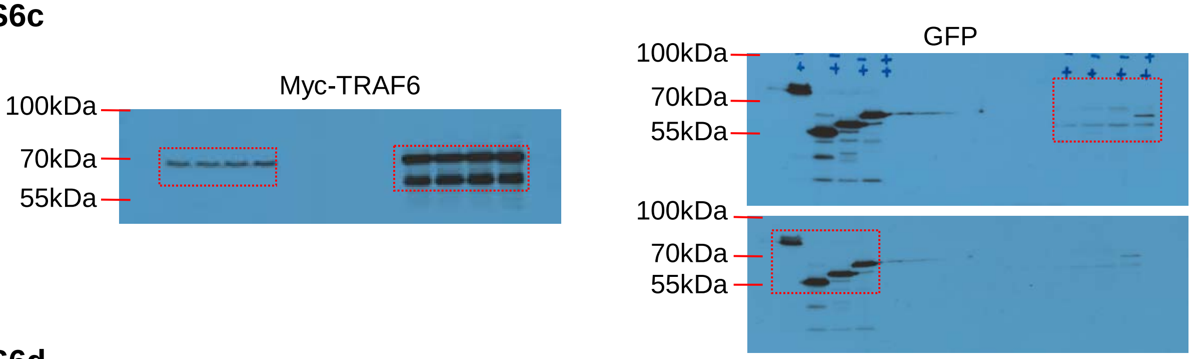

Fig. S6d

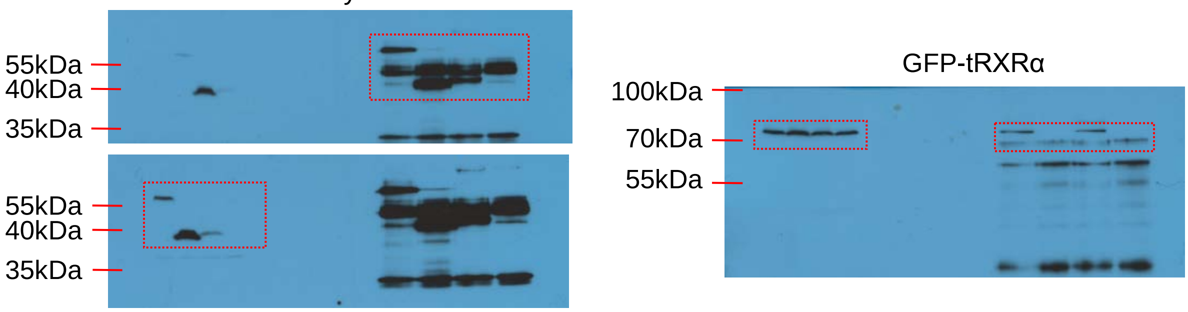

Fig. S6e

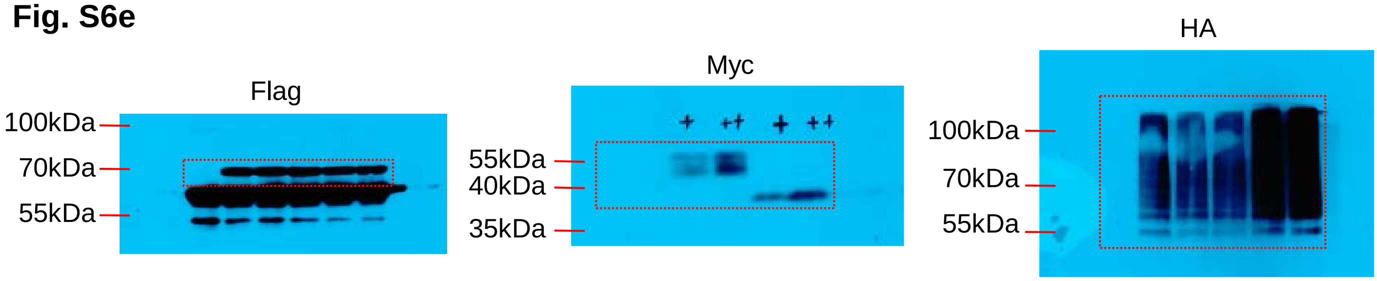

Fig. S6f

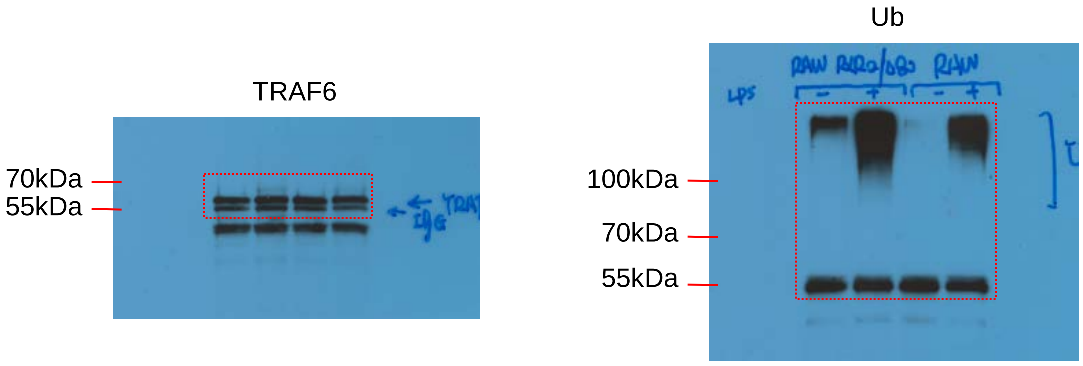

Fig. S7e

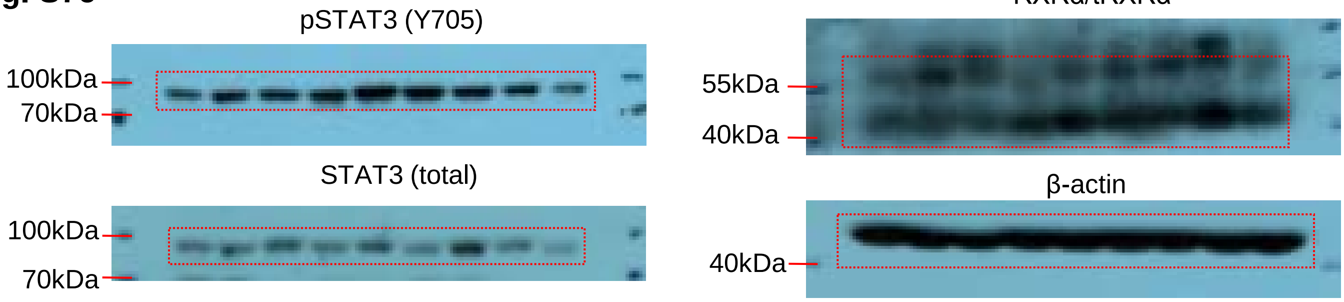

Fig. S7g

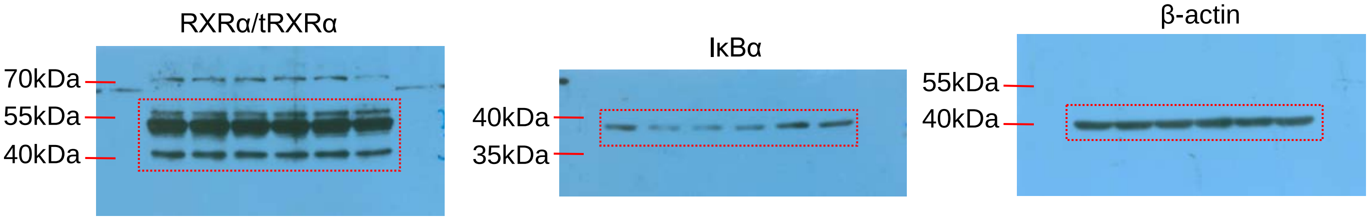

Fig. S7j

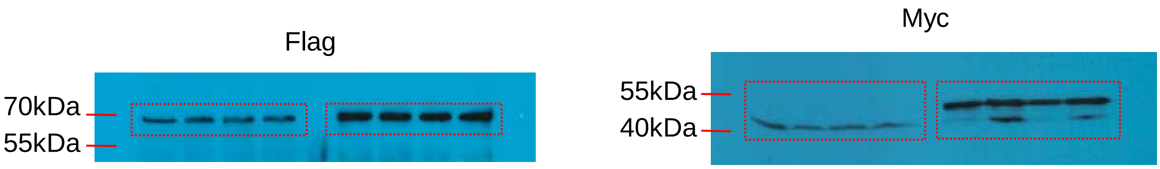

Supplementary Figure 8. Unprocessed scans of immunoblots shown in the main figures.
