# Supplementary material for: Oncogenic potential of truncated RXRα during colitis-associated colorectal tumorigenesis by promoting IL-6-STAT3 signaling
Source: Nat Commun. 2019 Apr 1;10:1463. doi: 10.1038/s41467-019-09375-8 (PMC6443775; doi:10.1038/s41467-019-09375-8)
Supplement: Supplementary file 2 — Description of Additional Supplementary Files [file 41467_2019_9375_MOESM2_ESM.pdf]

**Description of Additional Supplementary Files**

File Name: Supplementary Data 1
Description: Raw data for immunoblotting.
