# Supplementary material for: Oncogenic potential of truncated RXRα during colitis-associated colorectal tumorigenesis by promoting IL-6-STAT3 signaling
Source: Nat Commun. 2019 Apr 1;10:1463. doi: 10.1038/s41467-019-09375-8 (PMC6443775; doi:10.1038/s41467-019-09375-8)

# Supplementary Data 1

Ye et al.

Raw data for immunoblotting

Fig. 2a

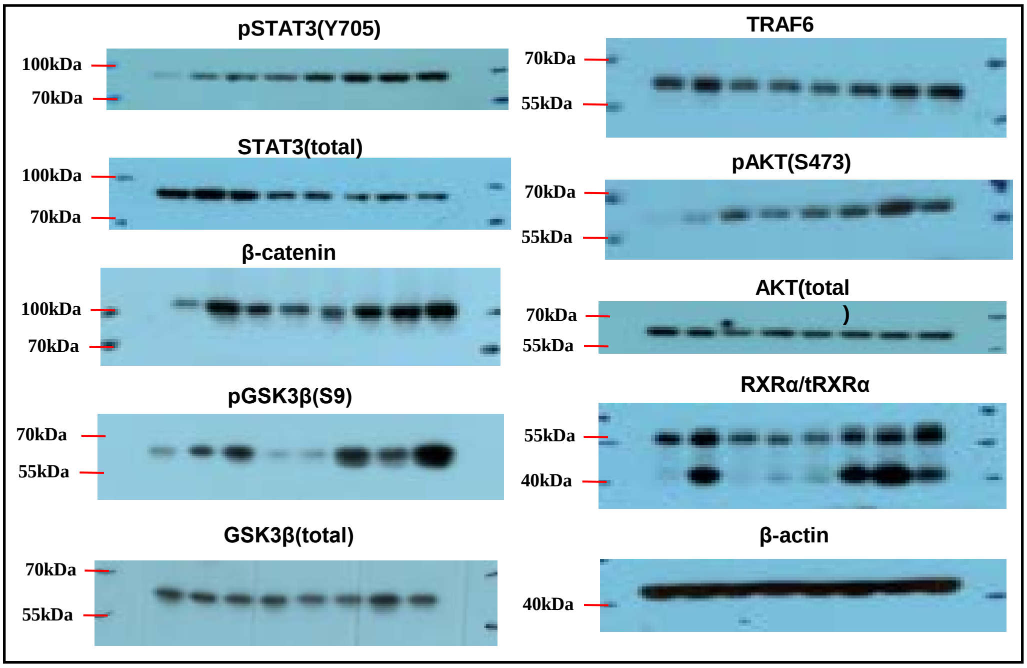


Fig. 2b

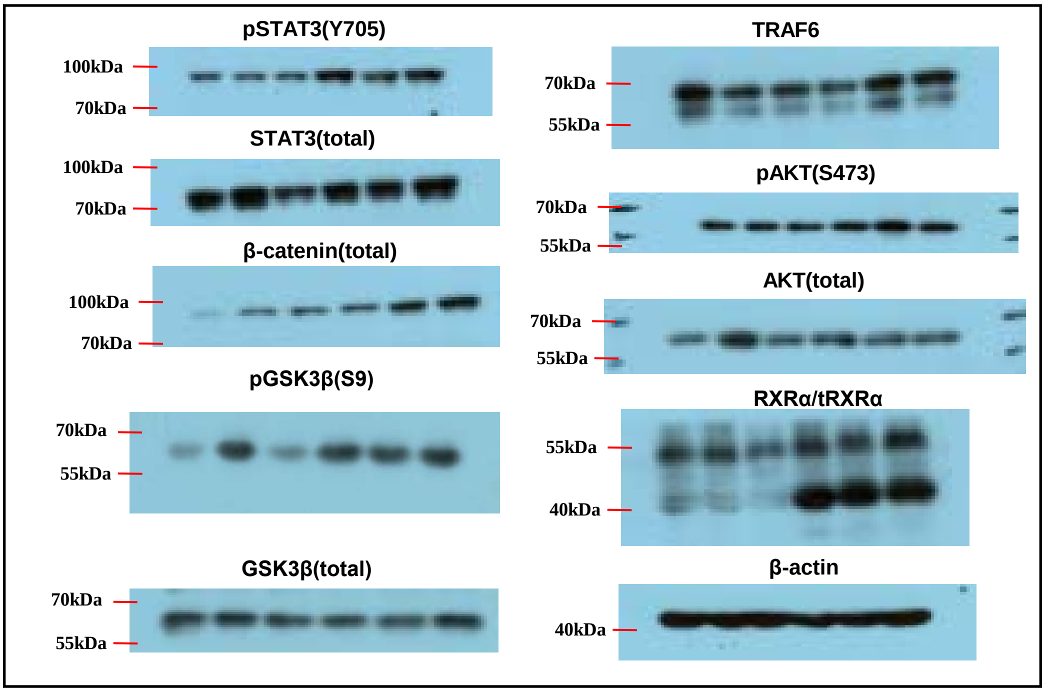


Fig. 2f

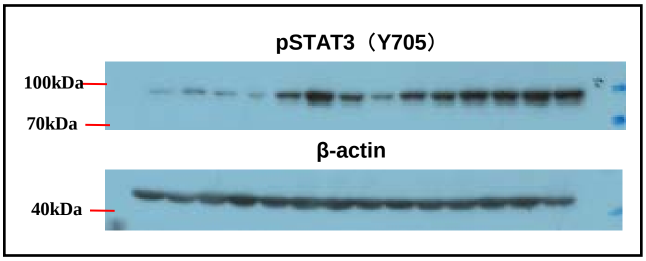

Fig. 4f

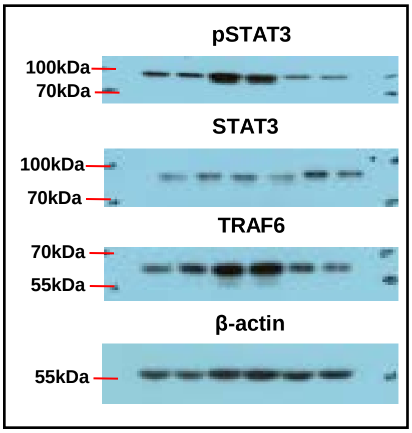


Fig. 5c

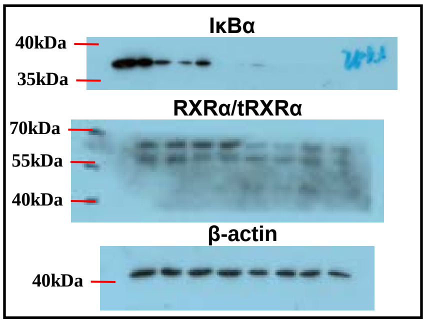


Fig. 5f

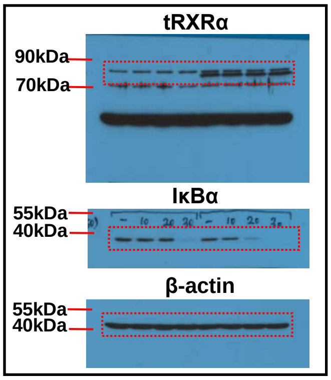


Fig. 5h

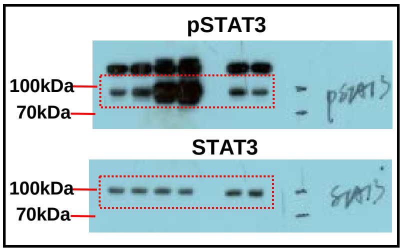


Fig. 6a

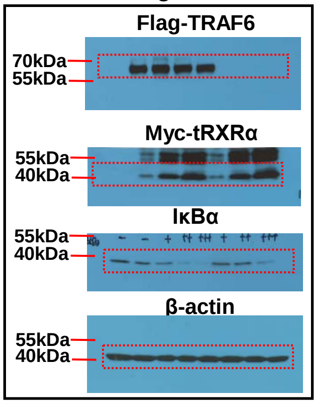


Fig. 6e

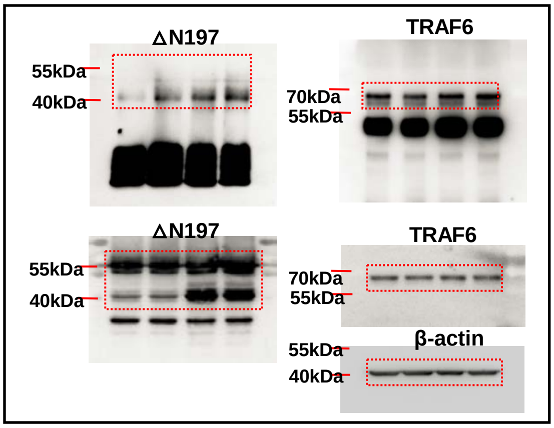


Fig. 6f

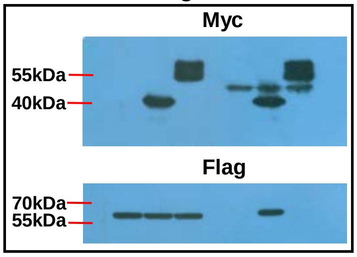


Fig. 6h

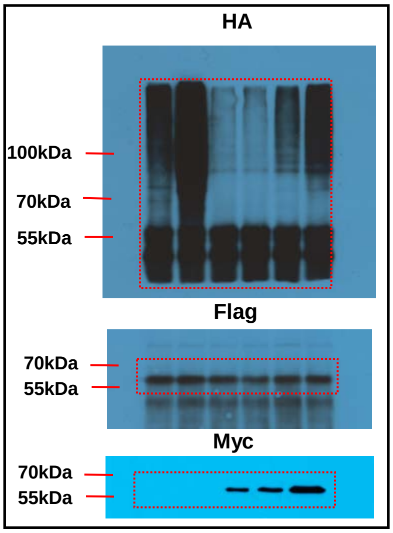


Fig. 6i

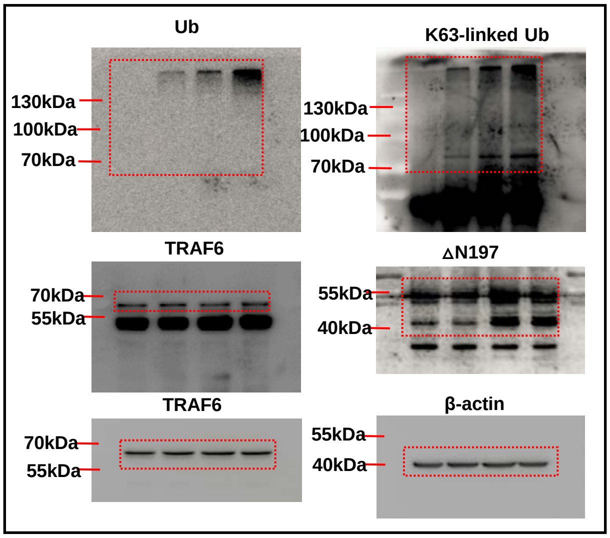


Fig. 6g

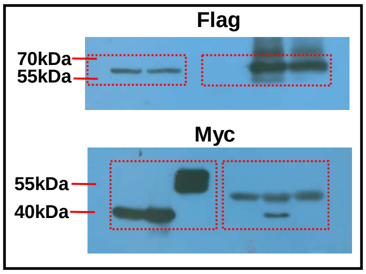

Fig. 7a

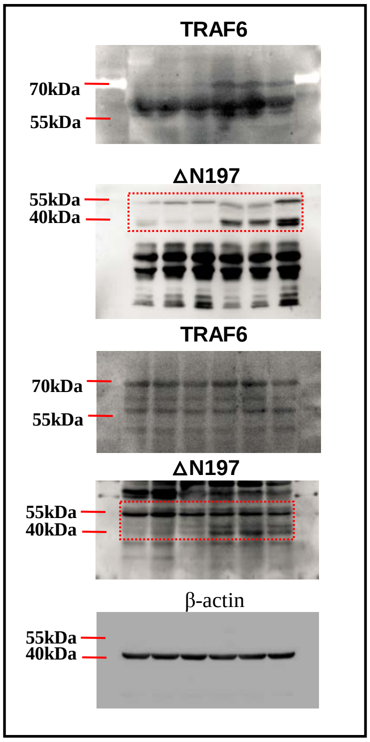


Fig. 7b

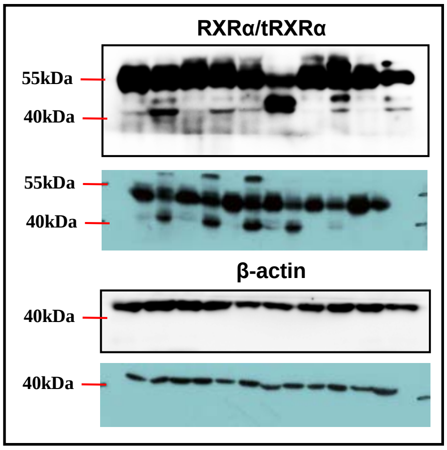


Fig. 7g

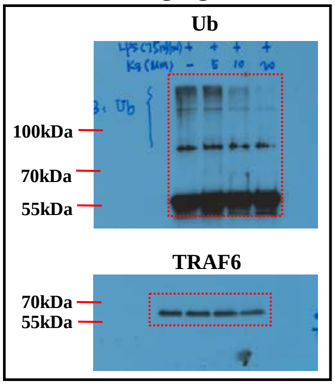

Fig. S1c

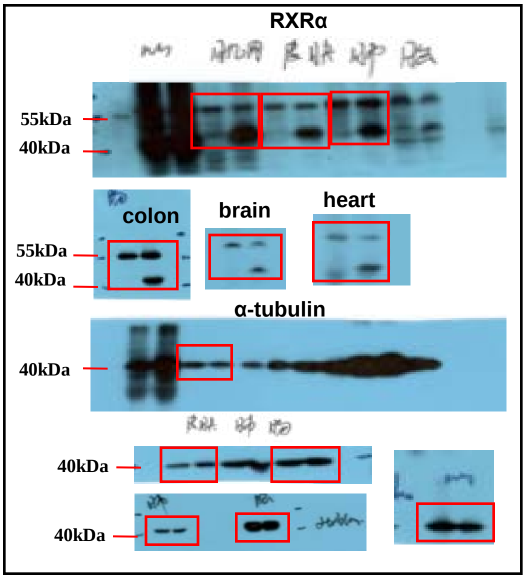


Fig. S4c

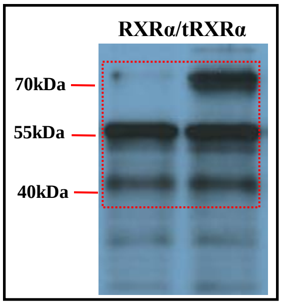


Fig. S4d

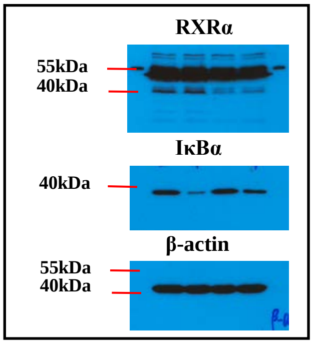


Fig. S5a

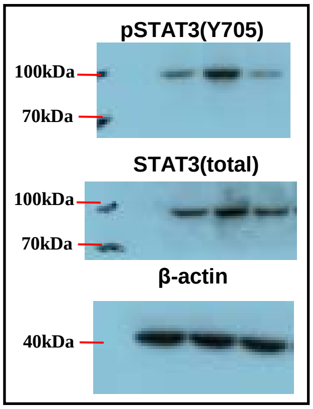


Fig. S5c

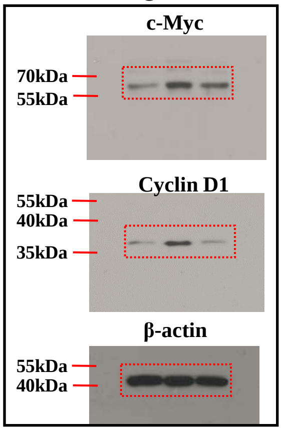


Fig. S6a/RAW264.7

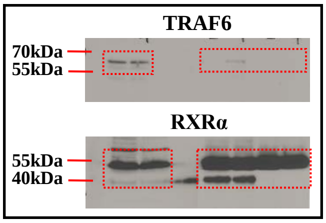


Fig. S6a/THP1

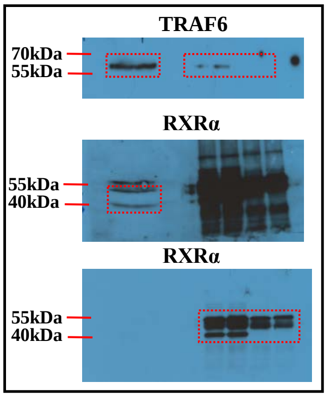


Fig. S6c

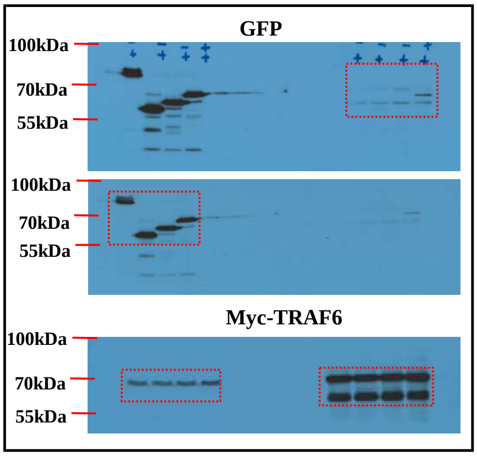


Fig. S6d

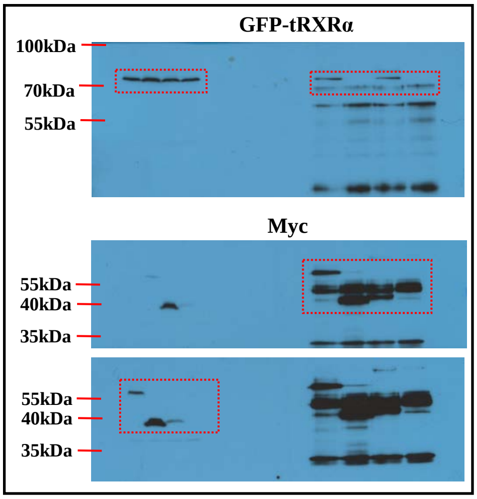

Fig. S6e

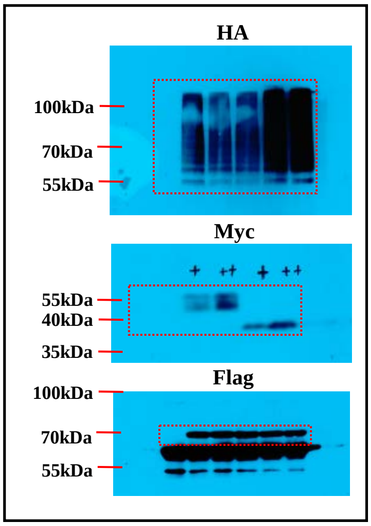


Fig. S6f

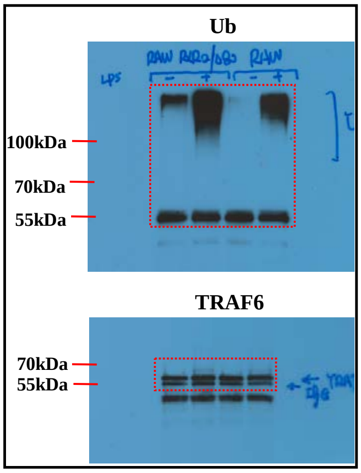


Fig. S7e

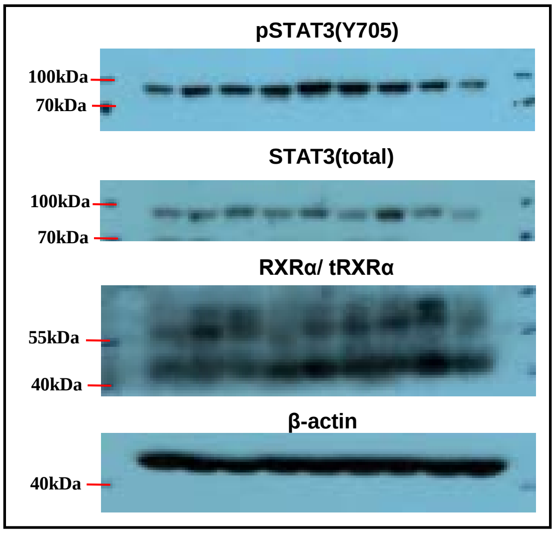


Fig. S7g

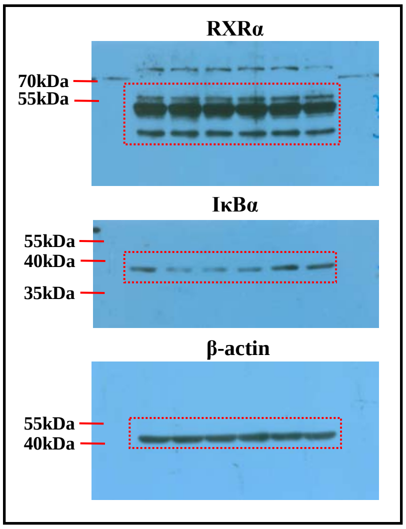


Fig. S7j

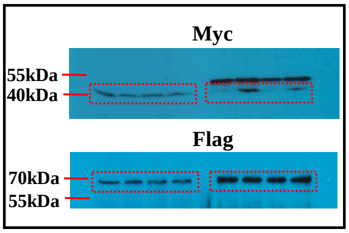

Supplement: Supplementary file 3 — Supplementary Data 1 [file 41467_2019_9375_MOESM3_ESM.pdf]
