# Supplementary material for: Oncogenic potential of truncated RXRα during colitis-associated colorectal tumorigenesis by promoting IL-6-STAT3 signaling
Source: Nat Commun. 2019 Apr 1;10:1463. doi: 10.1038/s41467-019-09375-8 (PMC6443775; doi:10.1038/s41467-019-09375-8)
Supplement: Supplementary file 4 — Reporting Summary [file 41467_2019_9375_MOESM4_ESM.pdf]

nature research

Corresponding author(s): Xiao-kun Zhang

Last updated by author(s): Jan 21, 2019

Reporting Summary

Nature Research wishes to improve the reproducibility of the work that we publish. This form provides structure for consistency and transparency in reporting. For further information on Nature Research policies, see Authors & Referees and the Editorial Policy Checklist.

Statistics

For all statistical analyses, confirm that the following items are present in the figure legend, table legend, main text, or Methods section.

n/a	Confirmed	
☐	☒	The exact sample size (*n*) for each experimental group/condition, given as a discrete number and unit of measurement
☐	☒	A statement on whether measurements were taken from distinct samples or whether the same sample was measured repeatedly
☐	☒	The statistical test(s) used AND whether they are one- or two-sided<br>*Only common tests should be described solely by name; describe more complex techniques in the Methods section.*
☒	☐	A description of all covariates tested
☒	☐	A description of any assumptions or corrections, such as tests of normality and adjustment for multiple comparisons
☐	☒	A full description of the statistical parameters including central tendency (e.g. means) or other basic estimates (e.g. regression coefficient) AND variation (e.g. standard deviation) or associated estimates of uncertainty (e.g. confidence intervals)
☐	☒	For null hypothesis testing, the test statistic (e.g. F, t, r) with confidence intervals, effect sizes, degrees of freedom and P value noted<br>*Give P values as exact values whenever suitable.*
☒	☐	For Bayesian analysis, information on the choice of priors and Markov chain Monte Carlo settings
☒	☐	For hierarchical and complex designs, identification of the appropriate level for tests and full reporting of outcomes
☒	☐	Estimates of effect sizes (e.g. Cohen's d, Pearson's r), indicating how they were calculated

Our web collection on statistics for biologists contains articles on many of the points above.

Software and code

Policy information about availability of computer code

Data collection	N/A
Data analysis	Graphs and charts data analyzed using GraphPad Prism.

For manuscripts utilizing custom algorithms or software that are central to the research but not yet described in published literature, software must be made available to editors/reviewers. We strongly encourage code deposition in a community repository (e.g. GitHub). See the Nature Research guidelines for submitting code & software for further information.

Data

Policy information about availability of data

All manuscripts must include a data availability statement. This statement should provide the following information, where applicable:

- Accession codes, unique identifiers, or web links for publicly available datasets
- A list of figures that have associated raw data
- A description of any restrictions on data availability

Provide your data availability statement here.

Field-specific reporting

Please select the one below that is the best fit for your research. If you are not sure, read the appropriate sections before making your selection.

☒ Life sciences ☐ Behavioural & social sciences ☐ Ecological, evolutionary & environmental sciences

For a reference copy of the document with all sections, see nature.com/documents/nr-reporting-summary-flat.pdf

Life sciences study design

All studies must disclose on these points even when the disclosure is negative.

Sample size	Sample size was determined to be adequate based on the magnitude and consistency of measurable differences between groups.
Data exclusions	On principle, data were only excluded for failed experiments, reasons for which included suboptimal activation and microbial contamination.
Replication	Replicate experiments were successful.
Randomization	No randomization of mice. Mice analyzed were litter mates and sex-matched whenever possible.
Blinding	Investigators were not blinded to mouse genotypes during experiments. Data reported for mouse experiments are not subjective.

Reporting for specific materials, systems and methods

We require information from authors about some types of materials, experimental systems and methods used in many studies. Here, indicate whether each material, system or method listed is relevant to your study. If you are not sure if a list item applies to your research, read the appropriate section before selecting a response.

Materials & experimental systems

n/a	Involved in the study	
☐	☒	Antibodies
☐	☒	Eukaryotic cell lines
☒	☐	Palaeontology
☐	☒	Animals and other organisms
☒	☐	Human research participants
☒	☐	Clinical data

Methods

n/a	Involved in the study	
☒	☐	ChIP-seq
☒	☐	Flow cytometry
☒	☐	MRI-based neuroimaging

Antibodies

Antibodies used	Anti-RXRα (D20) (Cat. sc-553), anti- RXRα (△N197) (Cat. sc-774), Anti-Myc (9E10) (Cat. Sc-40), anti TRAF6(D-10) (Cat. sc-8409), anti-TRAF6(H-274) (Cat. sc-7221), anti- AKT (Cat. sc-8312), anti-β-catenin(E-5) (Cat. sc-7963), anti-PCNA (FC-261) (Cat. sc7907) and anti-p65 (Cat. sc-109) antibodies were from Santa Cruz Biotechnology (Santa Cruz, CA, USA). Anti- IκBα (Cat. ab32518), anti-pSTAT3 (Y705) (Cat. ab76315), Anti-Ki-67 (Cat.ab15580) and anti-CD68 (Cat. ab31630) antibodies were from Abcam (UK). Anti-Ubiquitin (Cat. 3933S), anti-STAT3 (Cat. 9139S), anti-p-IκBα (Cat. 9246), anti-K63-ubiquitin (D7A11)(Cat.#5621), Anti-pGSK3β(Ser9)(Cat.#9336), and anti-pAKT(S473) (Cat. 9271S) antibodies were from Cell Signal Technology (Beverly, MA, USA). Anti-GSK3β(total)(Cat.610202) from BD biosciences (San Diego, USA). Anti-β-actin (Cat. A5441) and anti- FLAG (Cat. F1804) antibodies were from sigma (St. Louis, MO, USA).
Validation	More information in Methods section.

Eukaryotic cell lines

Policy information about cell lines

Cell line source(s)	RAW264.7, CV-1 cells, THP-1 and HEK293T were purchased from the American Type Culture Collection (Manassas, VA, USA). CT26.WT and HCT-116 were purchased form Cell Bank in Chinese Academy of Sciences in Shanghai.
Authentication	None of the cell lines used were authenticated.
Mycoplasma contamination	Cell lines were tested negative for mycoplasma.
Commonly misidentified lines (See ICLAC register)	No cell lines used are listed in the database of commonly misidentified cell lines.

Animals and other organisms

Policy information about studies involving animals; ARRIVE guidelines recommended for reporting animal research

Laboratory animals	Description of research mice used for experiments can be found in the relevant figure legends and Methods.
Wild animals	N/A

Field-collected samples	All of the mice were maintained with 12 h light/12 h dark cycles at the Laboratory Animal Center in Xiamen University.
Ethics oversight	All of the mice were complied with the Animal Care and Use Committee of Xiamen University

Note that full information on the approval of the study protocol must also be provided in the manuscript.
